# Oestrogen Receptor Alpha in Myocyte Maintains Muscle Regeneration in Duchenne Muscular Dystrophy

**DOI:** 10.1002/jcsm.13807

**Published:** 2025-04-21

**Authors:** Xiaofei Huang, Sijia Li, Huna Wang, Lei Zhao, Xihua Li, Shusheng Fan, Wanting Hu, Haowei Tong, Guangyao Guo, Dengqiu Xu, Luyong Zhang, Zhenzhou Jiang, Qinwei Yu

**Affiliations:** ^1^ New Drug Screening and Pharmacodynamics Evaluation Center China Pharmaceutical University Nanjing China; ^2^ Department of Neurology Children's Hospital of Fudan University Shanghai China; ^3^ Department of Hepatobiliary Surgery Innovative Institute of Tumor Immunity and Medicine (ITIM), Anhui Province key Laboratory of Tumor Immune Microenvironment and Immunotherapy The First Affiliated Hospital of Anhui Medical University Hefei China; ^4^ Center for Drug Research and Development Guangdong Pharmaceutical University Guangzhou China; ^5^ Key Laboratory of Drug Quality Control and Pharmacovigilance, Ministry of Education China Pharmaceutical University Nanjing China

**Keywords:** Duchenne muscular dystrophy, fulvestrant, oestradiol, oestrogen receptor alpha, skeletal muscle regeneration

## Abstract

**Background:**

Oestrogen receptor alpha (ERα) plays an important role in maintaining mitochondrial function and regulating metabolism in skeletal muscle. However, its alterations and potential mechanisms in Duchenne muscular dystrophy (DMD) remain incompletely understood. In this study, we demonstrated the protective role of ERα in myocyte for skeletal muscle regeneration in *mdx* mice and explored the therapeutic effects of oestrogen receptor modulators on DMD.

**Methods:**

DMD patients' biopsies were obtained for histological analysis to explore the expression of ERα. The phenotype of muscle was analysed by histology and molecular biology. The therapeutical effect of different oestrogen receptor modulators was examined in *mdx* mice treated with fulvestrant (FVT, 20 mg/kg once a week) or oestradiol (E2, 1 mg/kg per day) for 4 weeks. The protective effect of ERα was performed on *mdx* mice after conditional knockout of ERα in skeletal muscle (ERα^mKO^ *mdx* mice). Evidence of activation of ERα/oestrogen‐related receptor alpha (ERRα)/myogenic differentiation 1 (MyoD) signalling pathway was inspected in the primary myoblasts isolated from mice, and C2C12 cells received intervention with E2/FVT/*Esr1*‐siRNA/*Esrra* overexpression plasmid.

**Results:**

The ERα expression was increased in DMD patients' triceps (*p* < 0.05) and *mdx* mice muscles (*p* < 0.05). FVT reduced ERα levels in the *mdx* mice muscles (*p* < 0.01) but had no significant effect on skeletal muscle regeneration on *mdx* mice. Compared with *mdx* mice, E2 reduced the levels of creatine kinase (CK) and lactic dehydrogenase (LDH) (*p* < 0.001) in serum, enhanced skeletal muscle function, alleviated skeletal muscle atrophy and fibre loss and upregulated the expression of ERα in GAS (*p* < 0.001) and TA (*p* < 0.05). The myogenic factors such as myosin heavy chain (MyHC, *p* < 0.001), myogenin (MyoG, *p* < 0.05), MyoD (*p* < 0.05) and ERRα (*p* < 0.001) were increased in *mdx* mice GAS with E2. But E2 had no effect on ERα^mKO^ *mdx* mice. The primary myoblasts and C2C12 were treated with E2 displayed an increased‐on myocyte fusion index (*p* < 0.05), ERα MyoD and ERRα expressions (*p* < 0.05). The myocytes' fusion index (*p* < 0.05) and ERα, MyoD and ERRα expression (*p* < 0.05) were decreased in si‐*Esr1*‐transfected C2C12 cells and increased in OE‐*Esrra*‐transfected C2C12 cells.

**Conclusion:**

We demonstrated that ERα in myocyte exerted a protective effect on skeletal muscle regeneration in DMD patients and *mdx* mice through the ERα‐ERRα‐MyoD pathway, which has potential implications for DMD therapy strategies.

## 1 Introduction

Duchenne muscular dystrophy (DMD) is a progressive muscle wasting disease that is inherited by the X chromosome [1] and is typically characterized by abnormally elevated creatine kinase, muscle pseudohypertrophy, decreased myofibres with massive inflammatory cell infiltration and skeletal muscle fibrosis [2, 3]. The aetiology of DMD is a frameshift or nonsense mutation of the gene encoding dystrophin [4]. It is a part of the dystrophin‐associated protein complex (DAPC) that protects muscle cell membranes from physical damage during muscle contraction [5]. Gene therapy strategies for DMD have been actively pursued; although hindered by the heterogeneity of mutation types, some gene therapies have been approved for the treatment of specific types of DMD mutations. Currently, early use of glucocorticoids, which was capable on controlling severe pathological symptoms and prolonging patients' walking ability, is the standard clinical treatment for DMD [6]. However, they have serious side effects, including osteoporosis and metabolic dysfunction [7]. Therefore, finding small molecule drugs with stable efficacy and fewer side effect that can be applied to all mutation types in DMD patients is an important goal of current preclinical research.

Oestrogen, a steroid hormone that is primarily synthesized and secreted by the ovaries, plays a crucial role in regulating and maintaining the development of the reproductive system [8]. Moreover, oestrogen has diverse physiological functions in non‐reproductive tissues and organs, including the cardiovascular, nervous, immune and skeletal muscle systems [9]. Studies have shown that oestrogen receptors are widely expressed in skeletal muscle [10]. It has been determined that oestrogen influences skeletal muscle damage by reducing inflammation and oxidative stress, thereby affecting muscle regeneration [11]. This effect is mainly mediated by oestrogen and its receptors oestrogen receptor alpha (ERα) and oestrogen receptor beta (ERβ). Among them, ERα has received increased amounts of attention in this field. It has been reported that the excessive activation of ERα induces M1‐type polarization of macrophages to promote the release of the inflammatory factor interleukin‐6 (IL‐6) and tumour necrosis factor‐alpha (TNF‐α), leading to inflammation [12]. Oestrogen acts on ERα to reduce weight and fat accumulation [13]. Conversely, mice with skeletal muscle‐specific knockout of ERα exhibit increased fatigue and compromised contractile force in skeletal muscle [14].

DMD patients and *mdx* model mice present diverse cell populations within muscle tissue due to muscle damage [15]. The effects of ERα and oestrogen receptor modulators also exhibit variability across different cell types. Fulvestrant (ICI 182780, FVT) functions as an oestrogen receptor antagonist that primarily targets oestrogen receptors to inhibit oestrogen signalling [16]. Research has demonstrated that FVT, through oestrogen receptor blockade, mitigates the elevation of markers associated with skeletal muscle satellite cells induced by exercise and oestrogen [17]. Supplementation with oestradiol (E2) under conditions of muscle loss resulting from female ageing and menopause can induce satellite cell proliferation through targeted oestrogen receptor stimulation, thereby facilitating muscle repair [18]. Additionally, E2 has been shown to mitigate skeletal muscle damage caused by inflammatory stress [19]. By activating ERα in skeletal muscle cell mitochondria, E2 sustains glucose homeostasis and stabilizes oxidative metabolism in muscle fibres [20]. The effects of oestrogen receptor modulators on muscle function are intricate. It is evident that E2 exerts diverse beneficial effects on skeletal muscle, promoting muscle growth and repair. The promotion of myofibre formation is a critical factor in determining whether a drug or target can be considered a viable therapeutic strategy for DMD. We defined myoblast that have exited the cell cycle and initiated differentiation towards myofibres as a myocyte. The differentiation capacity of myocyte was critical for skeletal muscle regeneration and maturation in DMD. At this stage, MyoD in myocyte served as a myogenic factor marking the irreversible exit from the cell cycle, promoting myocyte differentiation and MyoG expression. Subsequently, the accumulation of MyoG facilitates the terminal differentiation of myocyte into myofibre [21, 22]. However, the mechanisms underlying ERα function in myocyte, as well as the distribution of ERα in myocyte of DMD patients or *mdx* mice, remain unclear.

Oestrogen‐related receptor alpha (ERRα) is a nuclear receptor that regulates gene transcription and translation and is widely present in various composite cells [23]. Studies have shown that ERRα also plays important physiological roles in skeletal muscle and that increasing the expression of ERRα in myocyte can promote myoblast differentiation and skeletal muscle growth and repair [24]. Therefore, we suppose that ERRα might be a co‐factor in ERα‐mediated skeletal muscle function regulation. In this study, we aimed to investigate the role of ERα under the pathological condition of DMD patients and *mdx* mice and explore the effect and mechanism of oestrogen receptor modulators on DMD via ERα/ERRα‐induced signalling pathway in vivo and in vitro.

## 2 Methods

### 2.1 Patients' Biopsies

All muscle biopsies were obtained from Children's Hospital of Fudan University. This protocol is registered clinical trial at Children's Hospital of Fudan University (NCT number: 2019‐244). All experiments based on clinical samples were approved by the Institutional Review Board at Children's Hospital of Fudan University (Shanghai, China), and all participants provided written informed consent prior to initiation of study procedures. Muscle biopsies as triceps brachii were obtained from healthy control with orthopaedic surgery and DMD patients matched by age (2–10 years, male). The study conformed to the ethical standards as set in the Declaration of Helsinki.

### 2.2 Animal Studies

All the experimental procedures were followed by the Animal Care and Use Committee of China Pharmaceutical University (Nanjing, China, 2021‐004‐008). All applicable international, national and/or institutional guidelines for the care and use of animals were followed (approval number: SCK (SU) 2018‐0019). *Dystrophin*‐deficient C57BL/10ScSnJ‐*mdx* mice (*mdx* mice) and C57BL/10ScSnJ mice (wild‐type mice, WT) were purchased from Jiangsu JicuiYaokang Biotechnology Co. Ltd. B6(Cg)‐*Esr1* ^*tm4.1Ksk*^/J‐*ERα* ^*lox*^ mice were purchased from Beijing Victory Lihua Laboratory Animal Technology Co. Ltd. B6. FVB (129S4)‐Tg (C*kmm‐cre*)5Khn/J mice were purchased from Shanghai Southern Model Biotechnology Co. Ltd. All animals were housed in individually ventilated cage systems (12 h chiaroscuro, standard mouse food and ad libitum). Three‐ to four‐week‐old WT male mice and *mdx* male mice [S1, S2] were randomly divided into WT and *mdx* groups were treated with a solvent (90% oil, 10% absolute ethanol) for subcutaneous injection, whereas the *mdx* FVT group was subcutaneously injected with 20 mg/kg fulvestrant (FVT, Selleck Cat# 129453‐61‐8, CHN) once a week for 4 weeks [16], or the *mdx* E2 group was subcutaneously injected with 1 mg/kg oestradiol benzoate (E2, Hangzhou Animal Medicine Factory Cat# 6930044700120) every day for 4 weeks [20]. B6(Cg)‐*Esr1* ^*tm4.1Ksk*^/J‐*ERα* ^*lox*^ mice were bred with *mdx* mice, respectively, and the offspring were purified to obtain homozygous ERα^fl/fl^ *mdx* mice. B6. Male FVB (129S4)‐Tg (*Ckmm‐cre*)5Khn/J mice were then bred with *mdx* female mice, and the offspring were subsequently purified to obtain homozygous *Ckmmcre*‐*mdx* mice. ERα^fl/fl^ *mdx* mice and *Ckmmcre*‐*mdx* mice were bred and purified to obtain homozygous ERα^mKO^ *mdx* mice. The *mdx* male mice and ERα^mKO^ *mdx* male mice groups were treated with a solvent (90% oil, 10% absolute ethanol) for subcutaneous injection, whereas the ERα^mKO^ *mdx* E2 group was subcutaneously injected with 1 mg/kg E2 every day for 4 weeks.

### 2.3 Histological Analysis

Mice were euthanized by inhalation of isoflurane, and the GAS, TA and DIA were harvested, immediately flash frozen in liquid nitrogen–cooled isopentane and stored at −80°C. Frozen muscles were sectioned at −20°C at a thickness of 10 μm and stained with haematoxylin and eosin (H&E) and Masson trichrome staining. The slides were viewed under a light microscope (BX53, Olympus, Japan).

### 2.4 Isolated Primary Culture of Mice Myoblasts

Primary myoblasts from WT mice and *mdx* mice (3–4 weeks old) were isolated as previously described. Briefly, the GAS muscles were washed with isotonic Dulbecco saline, minced into 1–2 mm^3^ fragments with eye scissors and digested with a collagenase/dispase/CacCl_2_ solution for 1.5 h at 37°C in a shaking bath. Then, the threefold diluted tissue lysate was passed through a mesh for cell suspension, precipitated and washed with isotonic Dulbecco saline. Cells were plated on an uncoated plate for differential plating. After 4 h, non‐adherent cells were seeded into a collagen‐coated flask in growth medium supplemented with DMEM supplemented with 20% FBS and 2.5 ng/mL β‐FGF (Gibco, Waltham, MA, USA). After the proliferation phase, the growth medium was replaced with fusion medium consisting of 2% horse serum (HS) (Gibco, Waltham, MA, USA). Cells were used at passages between 4 and 10 to maintain differentiation activity. The final DMSO concentration used in the myocyte experiments was always ≤ 1/1000. The control group also contained 1/1000 DMSO in each experiment.

### 2.5 Cell Differentiation and Transfection Assay

C2C12 cells were kindly provided by Stem Cell Bank, Chinese Academy of Sciences. For the cell differentiation assay, after the C2C12 reached approximately 80% confluence, the medium was changed to differentiation medium containing 1 μM FVT or 10 nM 17β‐oestradiol (E2, MedChemExpress Cat# 50‐28‐2) for 4 days [18]. In the experiment of transfecting siRNA sequences or overexpression plasmid into cells, when C2C12 reached 50% confluency, the medium is replaced with DMEM containing lipo3000 (Thermo Fisher Cat# L3000075) and si‐*Esr1* sequences (GenePharm Cat# 32870) or *Esrra* overexpression plasmid (GenePharm Cat# 5831). *Esr1* or *Esrra* mRNA via qRT‐PCR was detected after 24 h, and ERα or ERRα expression was detected via WB after 36–48 h of continued incubation. Differentiation medium (or differentiation medium containing E2) can be used to induce differentiation of C2C12 cells after 4 days.

### 2.6 Western Blotting

The excised GAS, TA and DIA muscles were immediately pulverized in liquid nitrogen and dissolved in RIPA lysis buffer (Beyotime, Shanghai, China) supplemented with 1% PMSF and protease and phosphatase inhibitors. Protein concentrations were determined by the BCA method. The proteins were mixed with Laemmli buffer containing dithiothreitol and boiled for 10 min. Total protein was fractionated on sodium dodecyl sulfate–polyacrylamide gels and transferred to nitrocellulose membranes (Millipore, Billerica, MA, USA) by electroblotting using a miniature transfer apparatus (Bio‐Rad Laboratories, Hercules, CA, USA). Next, the membranes were blocked with blocking buffer (Tris‐buffered saline containing 5% skim milk) for 1 h at room temperature. The membranes were then incubated overnight at 4°C with one of the following primary antibodies: anti‐GAPDH (1:1000; Santa Cruz Biotechnology Cat# sc‐32 233), anti‐MyHC (1:500; DSHB Cat# MF20), anti‐MyoD (1:500; DSHB Cat# D7F2), anti‐MyoG (1:1000; Santa Cruz Biotechnology Cat# sc‐12 732), anti‐ERα (1:1000; Affinity Cat# AF6058), anti‐ERRα (1:1000; Affinity Cat# DF7775) and anti‐PGC‐1α (1:1000; Proteintech Cat# 66369). Then, the membranes were incubated with the secondary antibody for 1 h, and signals were detected using a Bio‐Rad ECL system (Hercules, CA, USA). The bands were determined by densitometric analysis with ImageJ‐Pro Plus software.

### 2.7 Immunofluorescence

For immunofluorescence (IF), muscle cryosections or myoblasts mounted on a slide were fixed in 4% paraformaldehyde (PFA) for 15 min. Cryosections and cells were blocked in blocking buffer for 60 min. Then, the samples were incubated overnight with the following primary antibodies at 4°C: anti‐CD68 (1:200; BioLegend Cat# B165752), anti‐MyHC (1:50; DSHB Cat# MF20), anti‐eMyHC (1:500, DSHB Cat# F1.652), anti‐Laminin (1:200; Sigma‐Aldrich Cat# L9393) and anti‐ERα (1:1000; Affinity Cat# AF6058). Subsequently, the samples were incubated with the following secondary antibodies: anti‐rabbit IgG, Alexa Fluor 488 (1:500; Jackson ImmunoResearch Cat# 151170), anti‐rabbit Alexa Fluor 647 (1:500; Jackson ImmunoResearch Cat# 152230) and anti‐mouse Alexa Fluor 647 (1:500; Jackson ImmunoResearch Cat# 124191) for 1 h at room temperature. 4′,6‐Diamidino‐2‐phenylindole (DAPI) was used to visualize the nuclei. The sections were rinsed 3 times in PBS for 5 min each and viewed under a fluorescence microscope (FV3000, Olympus, Japan). IF was quantified by ImageJ.

### 2.8 Data and Statistical Analysis

The data were analysed by GraphPad Prism 8 and are presented as the means ± SDs. The group size (*n*) is described in the figure legends and refers to the number of independent values used in the statistical analysis. Results were shown at least three independence experiments. No data were excluded from the statistical analysis. Dunnett's *t*‐test, which is protected by copyright, was used for this study. All rights reserved. In comparisons between two groups, Dunnett's *t*‐test was used. For comparisons of multiple groups, one‐way with the Tukey–Kramer post hoc test was used. Post hoc tests were conducted only if F in ANOVA achieved significance, and there was no significant variance inhomogeneity. Values of *p* < 0.05 were considered to indicate statistical significance.

## 3 Results

ERα is upregulated in the skeletal muscle of DMD patients and *mdx* mice.

**FIGURE 1:**
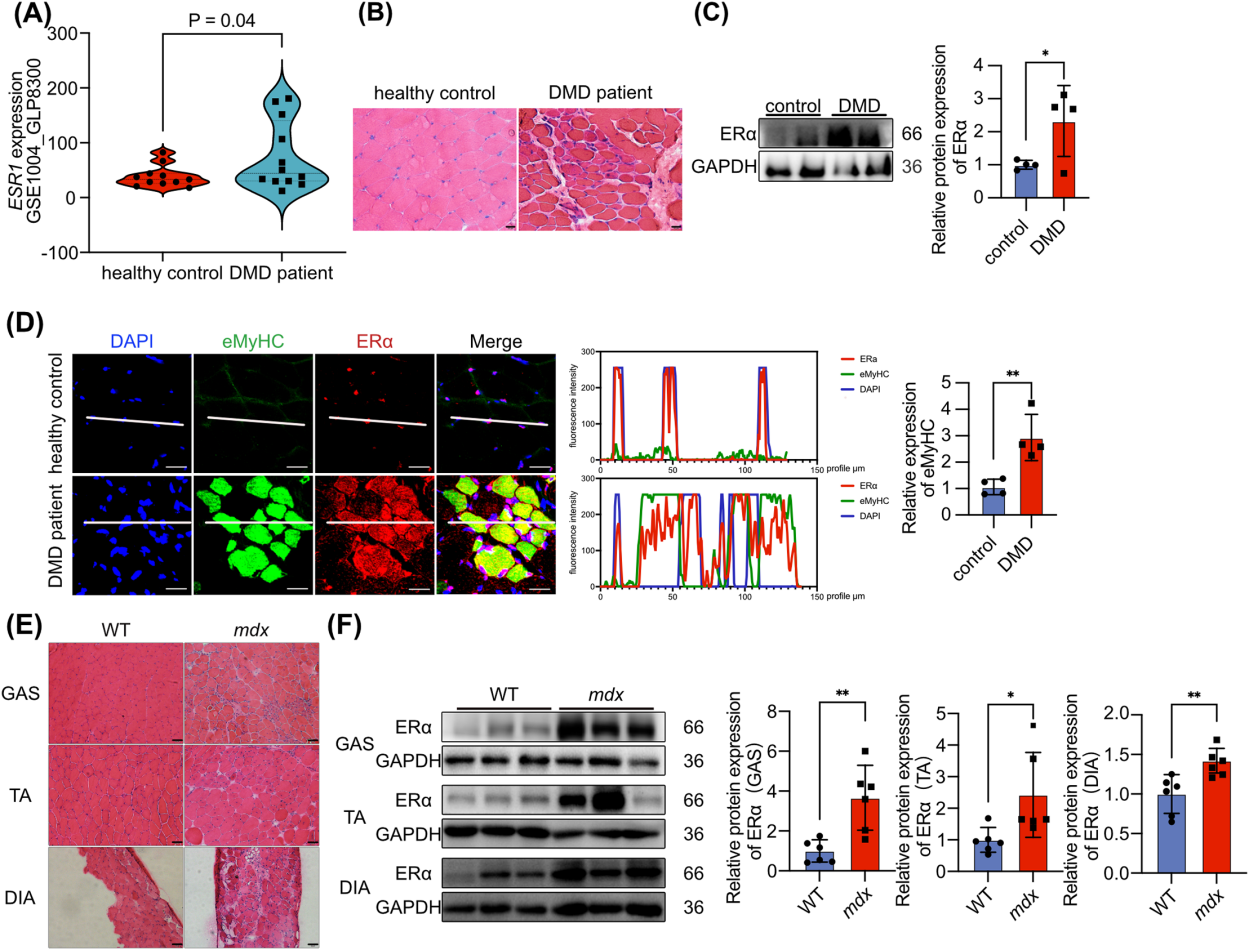
ERα is upregulated in the skeletal muscle of Duchenne muscular dystrophy patients and *mdx* mice. (A) Comparison of *ESR1* expression between DMD patients and healthy controls in the GSE1004_GLP8300 dataset (*p* = 0.04, healthy controls: *n* = 11; DMD patients: *n* = 12). (B) H&E staining of skeletal muscle from DMD patients and healthy people. Scale bar: 20 μm. (C) ERα protein expression in the skeletal muscle of DMD patients and healthy people (* vs. healthy controls, *p* < 0.05). (D) Immunofluorescence (IF) staining of ERα and eMyHC in the skeletal muscle of DMD patients and healthy people. Fluorescent co‐localization of ERα and eMyHC. Quantification of eMyHC expression. Scale bar: 25 μm. (E) H&E staining of mice muscle. Scale bar: 50 μm. (F) ERα protein expression in the GAS, TA and DIA muscles of mice. Eight‐ to ten‐week‐old males were used (*n* = 6). The data are presented as the means ± SDs; *p < 0.05 and **p < 0.01.

Studies have demonstrated the expression of ERα in muscle tissue, where it plays a critical role in regulating skeletal muscle mass and fibre type [25]. Continuous muscle damage in DMD leads to muscle fibre degeneration, inflammation and fibrosis [26]. To illuminate the alterations and impacts of ERα in DMD, we analysed DNA microarray data obtained from muscle quadriceps of DMD patients (aged 1–8 years) and healthy controls available in the GEO database and compared the *ESR1* levels. Our analysis suggested that the *ESR1* levels was upregulated in DMD patients (GSE1004_GLP8300 [27, 28]) (Figure 1A). To further confirm the changes in ERα in DMD patients, we examined the triceps brachii biopsy samples from DMD patients (aged 2–10 years) collected at Fudan Children's Hospital. Compared with healthy controls, histological analysis illustrated marked hypertrophy and the presence of numerous centralized nuclei in the muscle fibres (Figure 1B) and extensive infiltration of inflammatory cells in DMD patients (Figure S1A). We also demonstrated a significant increase in ERα expression and eMyHC expression in the muscle biopsy samples of DMD patients. The expression of eMyHC showed a distribution correlation with ERα (Figure 1C,D). To further study the role and specific molecular mechanisms of ERα in DMD, we employed *mdx* mice as the research model. We confirmed the absence of dystrophin in the skeletal muscle of *mdx* mice through IF staining (Figure S1B). We detected significantly elevated levels of CK and LDH in the serum (Figure S1C) and impaired behavioural functions in *mdx* mice, mainly manifested as reduced grip strength and hanging time (Figure S1D). The weight of the GAS in *mdx* mice was significantly increased, indicating GAS hypertrophy in *mdx* mice (Figure S1E). The cross‐sectional area (CSA) of muscle fibres in GAS, TA and DIA showed that *mdx* mice exhibit severe skeletal muscle atrophy (Figure S1G). The presence of centrally nucleated fibres indicated significant muscle damage in *mdx* mice (Figure S1F). IF staining of MyHC and eMyHC showed a reduction in mature muscle fibres in *mdx* mice, although there is some compensatory regeneration (Figure S1H,I). IF staining of CD68 revealed extensive infiltration of inflammatory cells in the skeletal muscle tissue of *mdx* mice (Figure S1J). IF staining of muscle fibre type indicated a reduction in the number of fast and slow muscle fibres in *mdx* mice (Figure S1K). Masson staining shows muscle fibre fibrosis in *mdx* mice (Figure S1M). IF staining of ERα and eMyHC in *mdx* mice skeletal muscle tissue indicated a correlation between ERα and the occurrence of newly formed muscle fibres, like results observed in DMD patients (Figure S1L). WB analysis implicated the upregulation of ERα in *mdx* mice muscles (Figure 1F). The above data clearly demonstrated that the pathological features and severity of *mdx* mice were like DMD patients and confirmed the upregulation of ERα in the skeletal muscle of both DMD patients and *mdx* mice.

### 3.1 The Oestrogen Receptor Antagonist FVT Partially Ameliorates Pathology in *mdx* Mice

**FIGURE 2:**
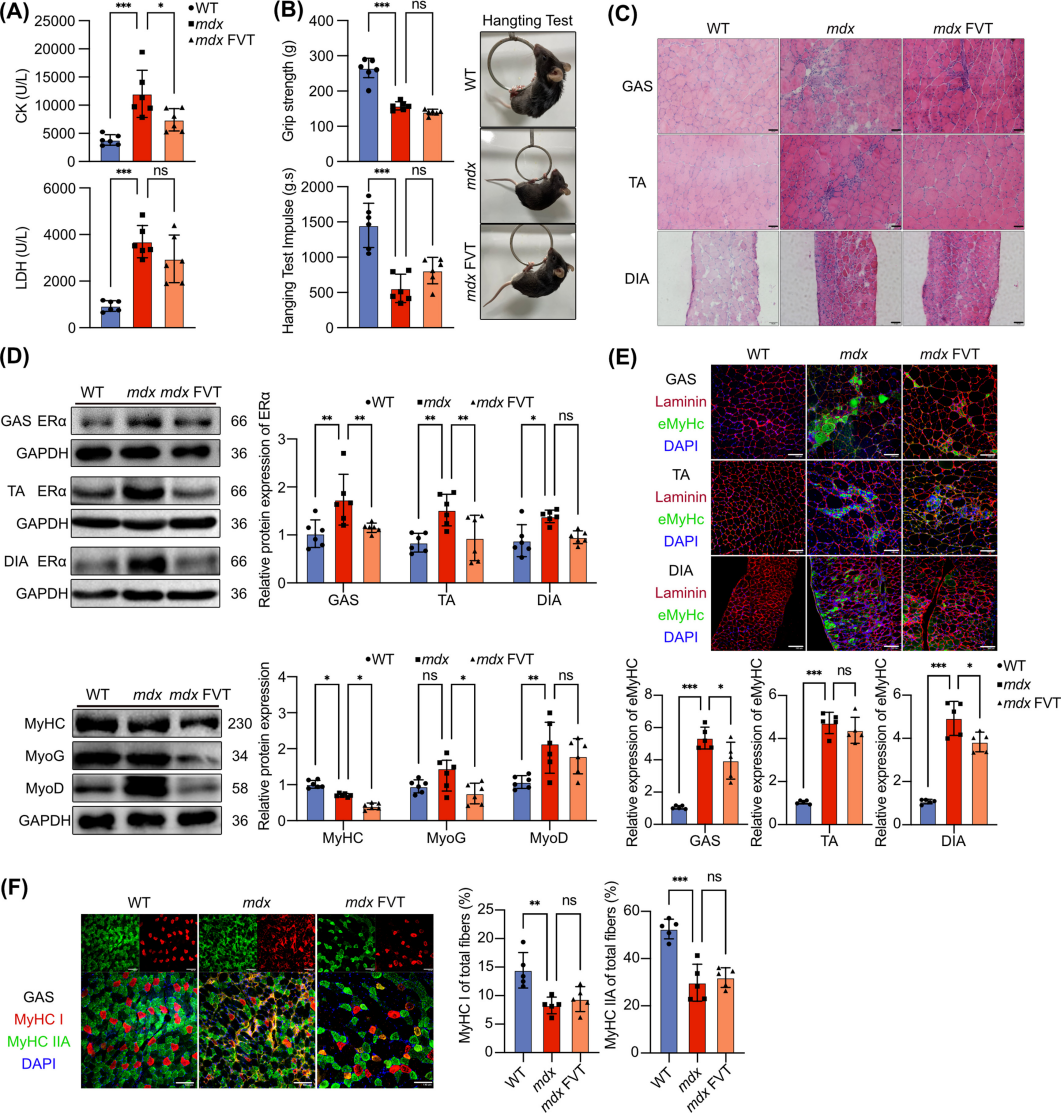
The oestrogen receptor antagonist FVT partially ameliorates pathology in *mdx* mice. (A) Contents of CK and LDH in mouse serum after treatment with FVT. (B) Grip strength and hanging test results for mice after treatment with FVT. (C) H&E staining of mouse muscle after treatment with FVT. Scale bar: 50 μm. (D) ERα protein expression in the GAS, TA and DIA muscles. Protein expression of MyHC, MyoG and MyoD in GAS after treatment with FVT. (E) IF staining of eMyHC and laminin in mouse muscle after treatment with FVT. Quantification of eMyHC expression. (F) IF staining of MyHC I and MyHC IIA in GAS after treatment with FVT. Quantification of MyHC I and MyHC IIA expression. IF staining scale bar: 100 μm. Eight‐ to ten‐week‐old males were used (*n* = 6). The data are presented as the means ± SDs; *p < 0.05, **p < 0.01, ***p < 0.001.

In both DMD patients and *mdx* mice muscles, ERα expression was upregulated. We hypothesized that downregulated ERα could improve the pathology of *mdx* mice. Therefore, we administered the oestrogen receptor antagonist FVT to *mdx* mice to investigate its therapeutic effects. FVT treatment did not significantly alter the body weight of the *mdx* mice (Figure S2A). FVT reduced serum CK levels in *mdx* mice but had no significant effect on lowering LDH levels (Figure 2A). Behavioural tests indicated that FVT did not significantly improve the recovery of grip strength or hanging time in *mdx* mice (Figure 2B). H&E staining, Masson's trichrome staining and IF staining of CD68 suggested that FVT partially alleviated skeletal muscle inflammation and fibrosis in *mdx* mice (Figures 2C and S2F,G). However, FVT did not significantly improve the skeletal muscle weight, centrally nucleated fibre occurrence, or CSA of muscle fibres in *mdx* mice (Figure S2B,D). WB analysis reflected that FVT indeed reduced ERα levels in *mdx* mice muscles consistent with existing research [16], with the most pronounced effect observed in the GAS. WB analysis of myogenic factors in GAS indicated that FVT downregulated the levels of myosin heavy chain MyHC, MyoG and MyoD in *mdx* mice muscles (Figure 2D). IF staining revealed a decrease in eMyHC expression in the skeletal muscle of *mdx* mice with FVT treatment, indicating impaired muscle regeneration. IF staining and WB analysis of MyHC demonstrated a persistent reduction mature muscle fibres in the skeletal muscle of *mdx* mice with FVT treatment, reflecting a decrease in the number of mature muscle fibres and unresolved muscle damage (Figures 2E,F and S2E). In summary, although FVT reduced ERα expression in *mdx* mice muscles, it did not significantly improve the pathology of *mdx* mice or restore skeletal muscle regeneration.

### 3.2 The Oestrogen Receptor Agonist E2 Ameliorates Pathological Symptoms of *mdx* Mice by Promoting Skeletal Muscle Regeneration

**FIGURE 3:**
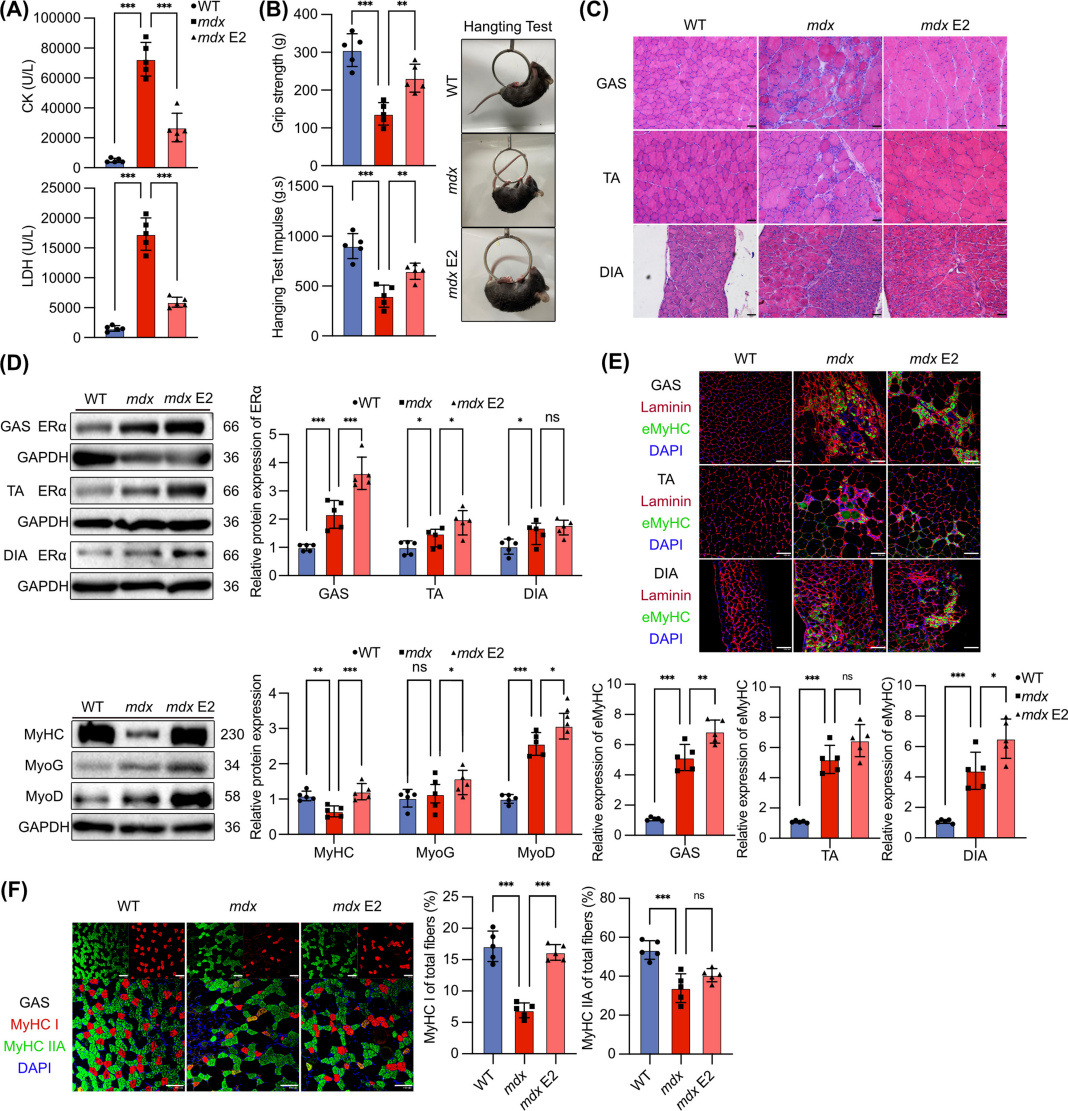
The oestrogen receptor agonist E2 ameliorates pathological symptoms in *mdx* mice promoting skeletal muscle regeneration. (A) Contents of CK and LDH in mouse serum after treatment with E2. (B) Grip strength and hanging test results for mice after treatment with E2. (C) H&E staining of mouse muscle after treatment with E2. Scale bar: 50 μm. (D) ERα protein expression in the GAS, TA and DIA muscles. Protein expression of MyHC, MyoG and MyoD in the GAS after treatment with E2. (E) IF staining of eMyHC and laminin in mouse muscle after treatment with E2. Quantification of eMyHC expression. (F) IF staining of MyHC I and MyHC IIA in GAS after treatment with E2. Quantification of MyHC I and MyHC IIA expression. IF staining scale bar: 100 μm. Eight‐ to ten‐week‐old males were used (*n* = 5). The data are presented as the means ± SDs; *p < 0.05, **p < 0.01, ***p < 0.001.

Previous studies have demonstrated the expression of ERα in various tissues and cells [29]. Administering FVT could serve to alleviate skeletal muscle inflammation by antagonizing ERα [30]. However, FVT reduced the expression of ERα in myocyte, suppressing myoblast differentiation and muscle fibre maturation. Consequently, we administered E2 to *mdx* mice to investigate whether it could exert therapeutic effects by activating ERα in myocyte. E2 treatment decreased the body weights and GAS weight of *mdx* mice (Figure S3A,B). Blood biochemical analysis indicated that E2 significantly reduced the levels of CK and LDH of *mdx* mice (Figure 3A). Grip strength and hanging time demonstrated that E2 enhanced skeletal muscle function in *mdx* mice (Figure 3B). H&E staining, central nuclei and CSA analysis showed that E2 alleviated skeletal muscle atrophy and fibre loss in *mdx* mice, restoring muscle fibre morphology (Figures 3C and S3C,D). Histological results illustrated that E2 reduced skeletal muscle fibrosis and inflammatory in *mdx* mice (Figure S3F,G). WB analysis indicated that E2 further increased ERα in *mdx* mice muscles, consistent with existing research [S3]. WB analysis proposed that E2 increased the expression of MyHC, MyoG and MyoD (Figure 3D). The results demonstrated that E2 increased the expression of MyHC and eMyHC and promoted the regeneration of newly formed muscle fibres and their differentiation into mature muscle fibres in *mdx* mice. E2 increased the number of different types of muscle fibres, particularly slow muscle fibres (Figures 3E,F and S3E). We detected ERα in both inflammatory cells and newly formed muscle fibres in *mdx* mice muscles (Figure S3H), indicating that ERα expressed in proliferating and differentiating myoblasts, as well as newly formed muscle fibres, played a protective role in skeletal muscles. E2 and FVT did not induce skeletal muscle pathological changes in wild‐type mice (Figure S2I). E2 could promote myoblast differentiation and skeletal muscle regeneration in *mdx* mice by activating ERα in myocyte.

### 3.3 ERα in Myocyte Activation Promotes Myoblast Differentiation and Myotube Formation

**FIGURE 4:**
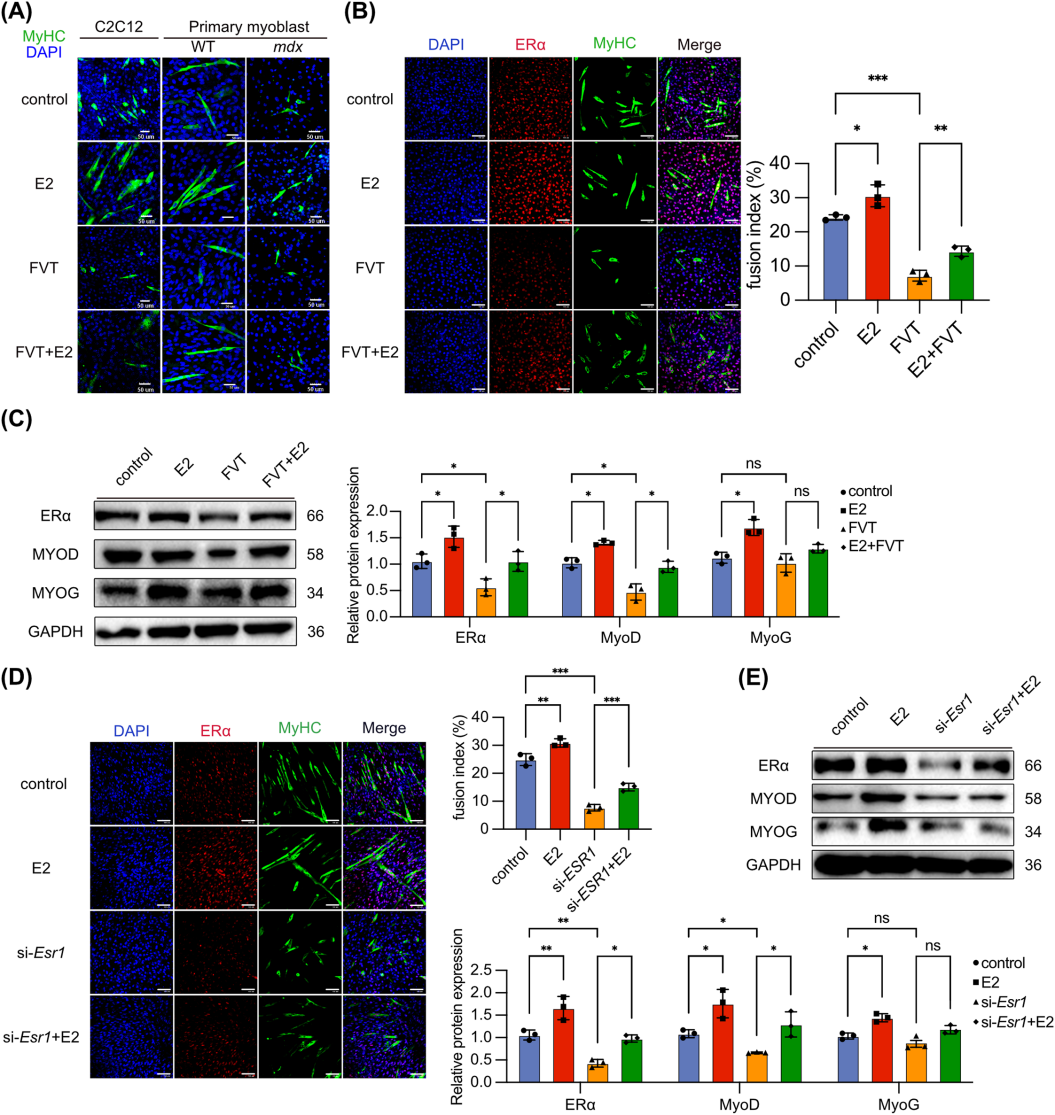
ERα activation promotes differentiation and myotubes formation of myocyte. (A) IF staining of MyHC on C2C12 cells and primary myoblasts from WT and *mdx* mice after administration of E2 and FVT. Scale bar: 50 μm. (B) IF staining of MyHC and ERα on C2C12 cells after administration of E2 and FVT and fusion index calculated as the average number of nuclei in MHC positive C2C12 cells. (C) Protein expression of ERα, MyoG and MyoD on C2C12 cells after the administration of E2 and FVT. (D) IF staining of MyHC and ERα on C2C12 cells after the transfection of si‐*Esr1* and the administration of E2 and fusion index calculated as the average number of nuclei in MyHC positive C2C12 cells. (E) Protein expression of ERα, MyoG and MyoD on C2C12 cells after the transfection of si‐*Esr1* sequence and the administration of E2. IF staining scale bar: 100 μm. The data are presented as the means ± SDs; *p < 0.05, **p < 0.01, ***p < 0.001.

To validate the important role of ERα in promoting skeletal muscle regeneration within myocyte, we isolated primary mice myoblasts. During the induction of differentiation in primary myoblasts from WT and *mdx* mice, E2 or FVT was administered. The results demonstrated that E2 facilitated myotube formation, whereas FVT hindered primary myoblast differentiation (Figure 4A). Consistent outcomes were observed in the myoblast cell line C2C12 (Figure 4B). WB analysis revealed that E2 upregulated ERα expression, augmented MyoD and MyoG expression and partially counteracted FVT‐induced differentiation inhibition (Figure 4C). The findings in C2C12 cells were consistent with results in *mdx* primary myoblasts (Figure S4A). To confirm the protective role of ERα in myocyte, we transfected small interfering RNA (siRNA) targeting *Esr1* into C2C12 cells and induced differentiation. *Esr1* was knocked down in si‐*Esr1*‐transfected C2C12 cells (Figure S4B). The ERα expression reduced in si‐*Esr1*‐transfected C2C12 cells, accompanied by decreased MyoD and MyoG expression and inhibition with myocyte fusion (Figure S4C,D). IF staining of MyHC indicated that E2 could promote myocyte fusion in si‐*Esr1*‐transfected C2C12 cells (Figure 4D). E2 increased the expression of ERα, MyoD or MyoG in si‐*Esr1*‐transfected C2C12 cells (Figure 4E). These findings demonstrated that *Esr1* knockdown in myocytes inhibited differentiation and confirmed that E2 acted by activating ERα in myocyte to promote myoblast differentiation and myocyte fusion.

### 3.4 E2 Targets ERα in Myocyte to Improve Skeletal Muscle Function in *mdx* Mice

**FIGURE 5:**
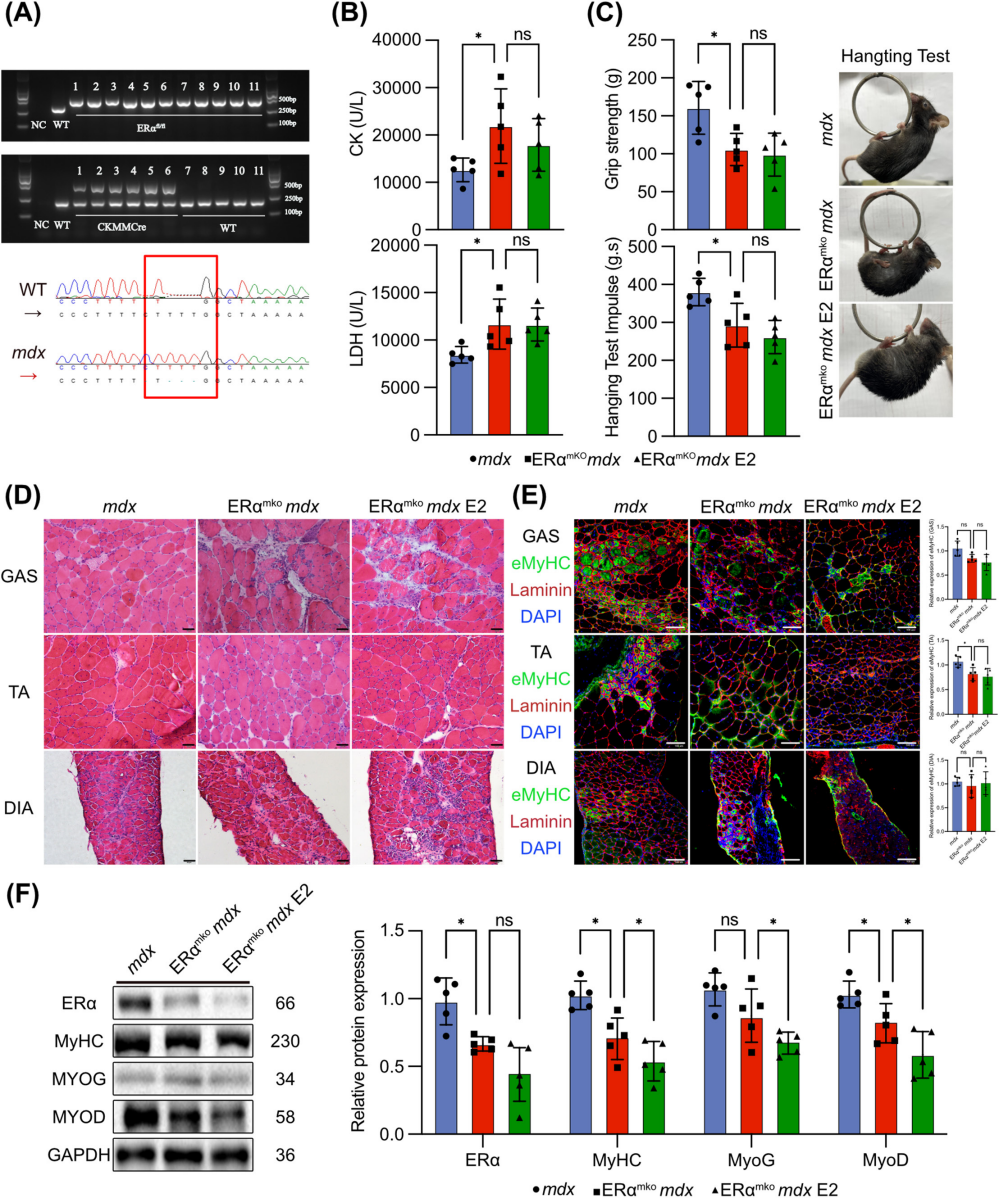
E2 targets ERα in myocyte to improve skeletal muscle function in *mdx* mice. (A) *Esr1* and *Ckmmcre* gene expression in WT mice and ERα^mKO^ *mdx* mice. Sequencing of the *Dystrophin* gene in WT and *mdx* mice. (B) Contents of CK and LDH in the serum of *mdx* mice, ERα^mKO^ *mdx* mice and ERα^mKO^ *mdx* mice after treatment with E2. (C) Grip strength and hanging test results for *mdx* mice, ERα^mKO^ *mdx* mice and ERα^mKO^ *mdx* mice after treatment with E2. (D) H&E staining of the muscle of *mdx* mice, ERα^mKO^ *mdx* mice and ERα^mKO^ *mdx* mice after treatment with E2. Scale bar: 50 μm. (E) IF staining of eMyHC and laminin in the muscle of *mdx* mice, ERα^mKO^ *mdx* mice and ERα^mKO^ *mdx* mice after treatment with E2. Quantification of eMyHC expression. Scale bar: 100 μm. (F) Protein expression of MyHC, MyoG and MyoD in the GAS of *mdx* mice, ERα^mKO^ *mdx* mice and ERα^mKO^ *mdx* mice after treatment with E2. Eight‐ to ten‐week‐old males were used (*n* = 5). The data are presented as the means ± SDs; *p < 0.05.

To further elucidate the protective role of ERα in myocyte, we generated *mdx* mice with skeletal muscle‐specific conditional knockout of ERα (ERα^mKO^ *mdx* mice) and treated with E2. First, we confirmed the successful establishment of ERα^mKO^ *mdx* mice through gel electrophoresis and conducted genotype sequencing to *Dystrophin* (Figure 5A). WB results demonstrated significant reduction of ERα expression in ERα^mKO^ *mdx* mice muscles (Figure S5A). Compared with *mdx* mice, the serum CK and LDH levels of ERα^mKO^ *mdx* mice were elevated, and E2 failed to alleviate this effect (Figure 5B). Similarly, a worsening of limb grip strength and hanging time in ERα^mKO^ *mdx* mice that E2 did not exert a therapeutic effect on (Figure 5C). There was no change in muscle weight in ERα^mKO^ *mdx* mice (Figure S5B). There were no significant changes in the CSA of the muscle or the occurrence of centrally nucleated fibres in ERα^mKO^ *mdx* mice, E2 even worsening skeletal muscle fibrosis (Figures 5D and S5B–E). IF staining indicated MyHC and eMyHC reduction, and a decrease in fast and slow myofibres in ERα^mKO^ *mdx* mice muscles even treated with E2 (Figures 5E and S5F,H). IF staining showed that infiltration of inflammatory cells was not reduced in ERα^mKO^ *mdx* mice muscles even treated with E2 (Figure S5G). WB of myogenic factor expression revealed a decrease in MyHC, MyoG and MyoD expression in ERα^mKO^ *mdx* mice muscles, along with a decrease in ERα, and E2 further decreased the expression of myogenic factors (Figure 5F). These findings collectively suggested that compared to *mdx* mice, ERα^mKO^ *mdx* mice exhibited more severe pathological conditions. Intriguingly, E2 did not ameliorate skeletal muscle regeneration defects in ERα^mKO^ *mdx* mice.

### 3.5 E2‐Mediated Skeletal Muscle Regeneration via ERα/ERRα/MyoD Pathway in Myocyte

**FIGURE 6:**
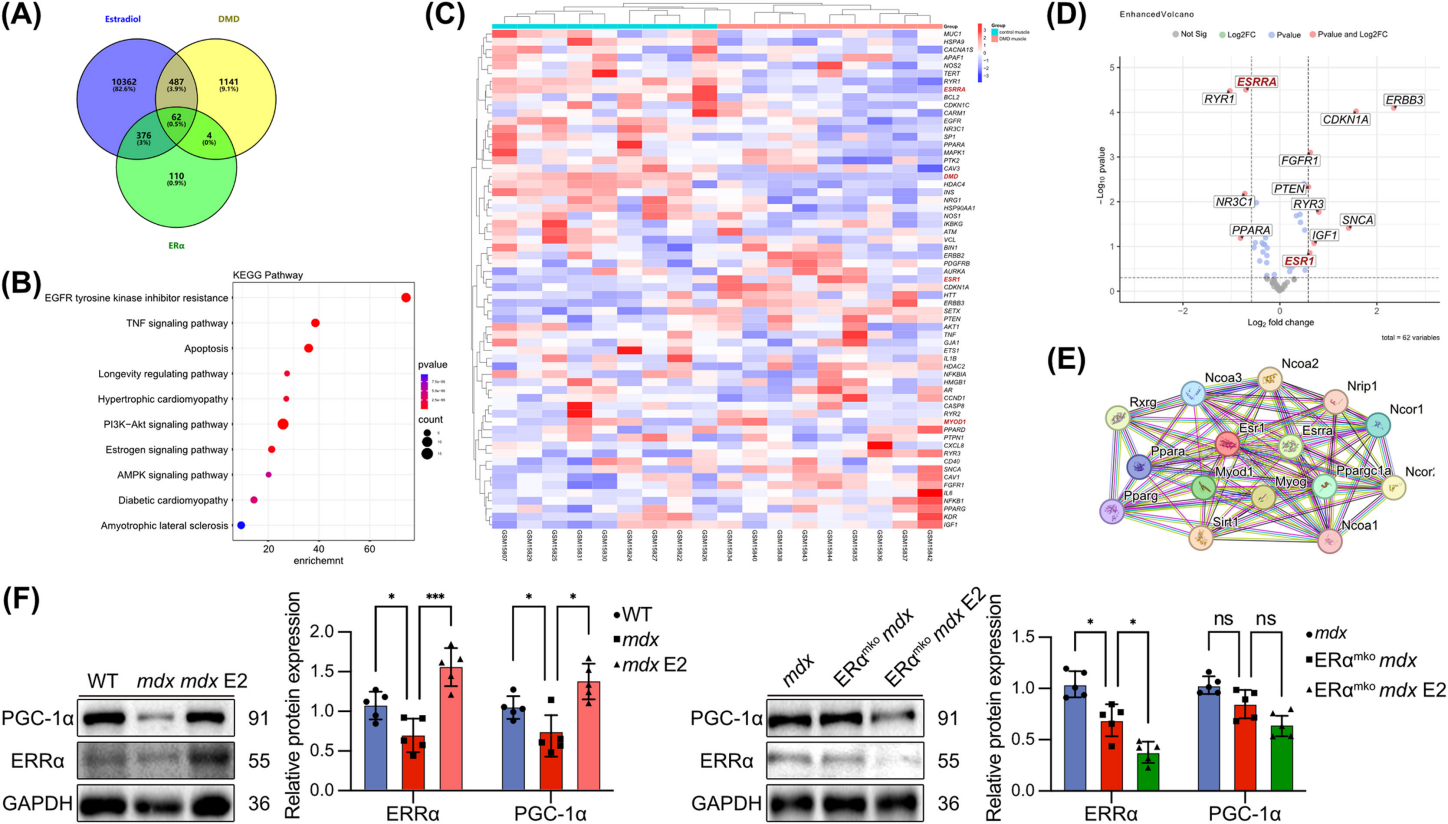
ERα‐mediated skeletal muscle regeneration by upregulating in DMD. (A) Venn diagram of intersecting genes related to E2 activity, DMD disease targets and ERα‐related targets. (B) Significant KEGG pathways of intersecting genes. (C) Heatmap of the expression of intersecting genes in DMD patients and healthy controls from GSE1004_GLP8300. (D) Volcano plot of intersecting genes in DMD patients and healthy people from GSE1004_GLP8300. E. PPI network constructed with intersecting genes. (F) Protein expression of ERRα and PGC‐1α in WT, *mdx* and *mdx* mice after treatment with E2. Protein expression of ERRα and PGC‐1α in *mdx* mice, ERα^mKO^ *mdx* mice and ERα^mKO^ *mdx* mice after treatment with E2. The data are presented as the means ± SDs; *p < 0.05, **p < 0.01, ***p < 0.001.

**FIGURE 7:**
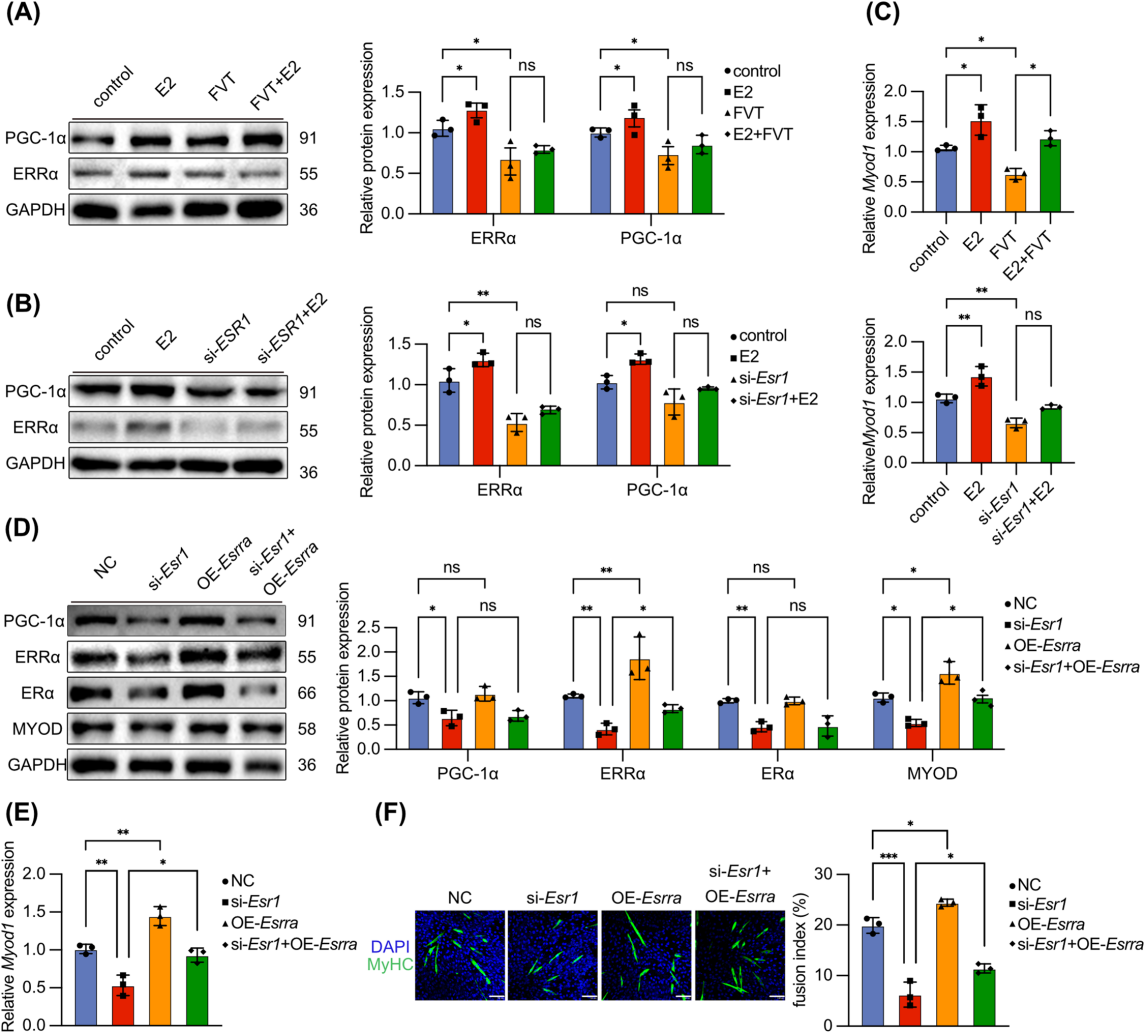
ERα‐mediated skeletal muscle regeneration via the ERα/ERRα/MyoD pathway in myocyte. (A) Protein expression of ERRα and PGC‐1α in C2C12 cells after the administration of E2 and FVT. (B) Protein expression of ERRα in C2C12 cells after the transfection of si‐*Esr1* and the administration of E2. (C) mRNA expression of *Myod1* on C2C12 cells after administration of E2, FVT, si‐*Esr1* or E2. D. Protein expression of ERRα, PGC‐1α, ERα and MyoD on C2C12 cells after the transfection of si‐*Esr1* and OE‐*Esrra*. (E) mRNA expression of *Myod1* on C2C12 cells after the administration of si‐*Esr1* and OE‐*Esrra*. (F) IF staining of MyHC on C2C12 cells after the transfection of si‐*Esr1* and OE‐*Esrra* and fusion index calculated as the average number of nuclei in MyHC positive C2C12 cells. IF staining scale bar: 100 μm. The data are presented as the means ± SDs; *p < 0.05, **p < 0.01.

Our research indicated that ERα in myocyte played a crucial role in promoting skeletal muscle regeneration in *mdx* mice treated with E2. However, the precise mechanism by which ERα facilitates myoblast differentiation remains unclear. Therefore, we sought to gain further insights into the underlying molecular mechanisms involved. The oestradiol dataset was analysed for potential biological targets using the Therapeutic Target Database and PubChem database. This analysis aimed to identify biological targets that oestradiol may affect. The DMD dataset, sourced from the MalaCards database, was utilized to enrich targets with altered expression levels during the progression of DMD. The ERα dataset, obtained from the NCBI‐PubChem database, focused on targets upstream and downstream of ERα. The intersections of these three datasets identified 62 intersecting genes (Figure 6A). GO enrichment analysis revealed the association with the oestrogen signalling pathway (Figure 6B). Comparative analysis between DMD patients and healthy controls identified 4 downregulated genes and 8 upregulated genes (GSE1004_GLP8300 [27, 28]) (Figure 6C,D). Through protein–protein interaction (PPI) network analysis, we selected ERRα as the target gene most strongly correlated with *Esr1* and *Myod1* among the 12 significantly altered DEGs [S4] (Figure 6E). Bioinformatics analysis and existing research have suggested that ERRα also played a role in myoblast differentiation. Studies have shown that ERRα forms heterodimers with PGC‐1α in various tissues, thereby participating in transcriptional regulation and influencing various biological functions [31]. Therefore, we hypothesized that in *mdx* mice muscles, E2 activates ERRα downstream of ER and, together with PGC‐1α, participates in the transcriptional regulation of MyoD, promoting skeletal muscle regeneration. We analysed the downregulation of *ESRRA* and *PPARGC1A* in DMD patients compared with healthy control (GSE1004_GLP8300 [27, 28], Figure S6C; GSE38417 [32], Figure S6D). We confirmed the downregulation of ERRα in *mdx* mice GAS and demonstrated that E2 increased the expression of ERRα and PGC‐1α. However, in ERα^mKO^ *mdx* mice muscles, the expression of ERRα was further decreased, and the expression of PGC‐1α was not decreased significantly. E2 did not promote the expression of ERRα and PGC‐1α (Figure 6F). We examined the expression of ERRα in C2C12 myoblasts treated with E2 or si‐*Esr1* interference. The results showed that E2 upregulated ERRα and PGC‐1α in C2C12 cells, whereas FVT inhibited (Figure 7A). The expression of ERRα and PGC‐1α decreased in si‐*Esr1*‐transfected C2C12 cells, and their expression could not be restored with E2 (Figure 7B). These results indirectly indicated that E2 activated the expression of ERRα by activating ERα. E2 promoted MyoD transcription in C2C12 cells, whereas *Myod1* levels decreased in si‐*Esr1*‐transfected C2C12 cells (Figure 7C). To verify the upstream–downstream relationship between ERα and ERRα, we transfected C2C12 with the *Esrra* overexpression plasmid (OE‐*Esrra*) and si‐*Esrra*. *Esrra* mRNA was increased in OE‐*Esrra*‐transfected C2C12 cells (Figure S6A). WB analysis indicated the increase expression of ERRα in OE‐*Esrra*‐transfected C2C12 cells, whereas ERα expression remained unchanged (Figure S6B). WB analysis revealed that, compared to the si‐*Esr1*‐transfected group, OE‐*Esrra* selectively upregulated ERRα, but not ERα or PGC‐1α (Figure 7D). Furthermore, OE‐*Esrra* partially rescued the *Myod1* reduction induced by si‐*Esr1* (Figure 7E). IF staining showed that OE‐*Esrra* promoted myocyte fusion and partially reversed the inhibition of myotube formation caused by si‐*Esr1* (Figure 7F). And we transfected C2C12 with si‐*Esrra*, decreasing the expression of *Esrra* mRNA (Figure S7A). WB analysis showed that si‐Esrra transfection did not decrease the expression of ERα (Figure S7B,C). This suggested that ERRα was downstream of ERα.WB and IF staining analysis indicated that si‐*Esrra* significantly decreased the protein levels of ERRα in C2C12 cells, leading to a reduction in MyoD and MyoG protein levels and inhibiting differentiation. E2 partially rescued the differentiation capacity of C2C12 cells inhibited by si‐*Esrra* (Figure S7B–E). The results of CHIP confirmed that *Myod1* promoter had a greater enrichment amount at ERRα compared to IgG. Moreover, E2 treatment significantly increased the binding of ERRα to the *Myod1* promoter compared to the untreated control group, which indicated that ERRα was recruited to this promoter region in response to E2 (Figure S7F). Overall, E2 upregulated ERRα via ERα in myocyte, thereby promoting MyoD transcriptional regulation, leading to enhanced myoblast differentiation and myotube formation.

## 4 Discussion

**FIGURE 8:**
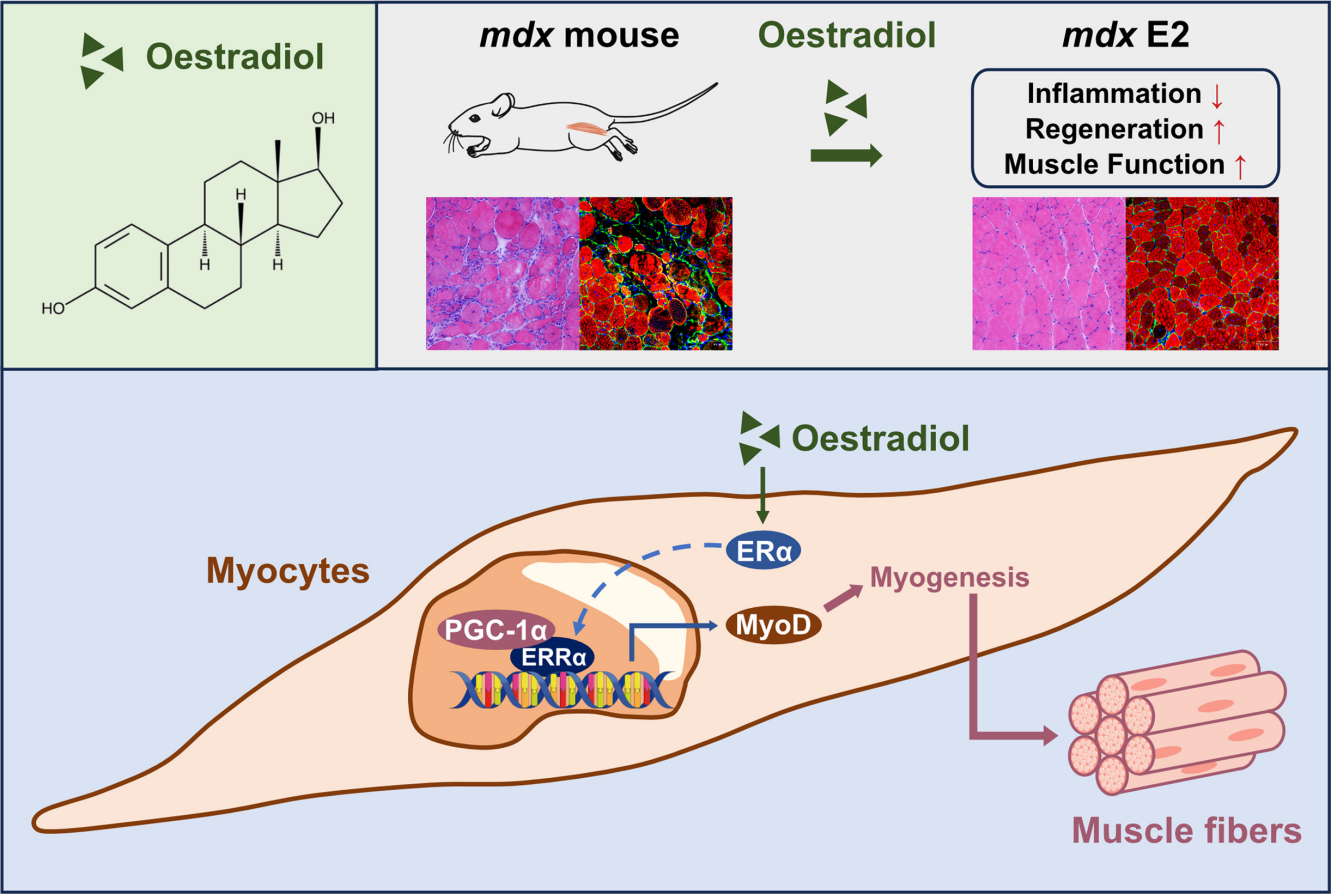
The signalling pathway of ERα protective effect in myocyte on DMD.

In this study, we verified the upregulation of ERα in both DMD patients and *mdx* mice muscles and the protective effect of ERα in myocyte on DMD by promoting skeletal muscle regeneration. The oestrogen receptor agonist E2 could enhance skeletal muscle regeneration and fibre repair by activating ERα/ERRα/MyoD signalling pathway. The mechanisms of ERα and the modulators in skeletal muscle suggested that targeting the activation of ERα in myocyte could potentially offer therapeutic benefits for DMD, thereby presenting novel avenues for drug research targeting DMD (Figure 8).

Oestradiol has known side effects in males, such as androgen antagonism, suppression of testosterone secretion and a reduction in secondary sexual characteristics. Prolonged oestradiol use (approximately 2 years) in men can result in the loss of sexual function [33]. DMD is an X‐linked muscle disease primarily affecting males; predictably, the use of oestradiol may have adverse effects on DMD patients in clinical. Here, E2 was used to initially validate that ERα could be a potential therapeutic target for DMD and to confirm that activation of ERα promoted skeletal muscle regeneration and improved the therapeutic value of ERα/ERRα agonist for DMD needed to be emphasized. Future research could choose more selective ERα agonists already used in treatments for osteoporosis [S5]. Developing ERα‐selective agonists with skeletal muscle tissue specificity would help avoid systemic side effects mediated by ERα activation, thus advancing the development of small molecule drugs for skeletal muscle regeneration in DMD.

ERα is involved in various biological processes by regulating the transcriptional activity of target genes and is expressed in a range of tissues and cells [13]. In this study, both in vivo and in vitro experiments, we used *mdx* mice with myocyte‐ and muscle tissue‐specific knockdowns, allowing us to specifically investigate the protective role of ERα in myocyte on skeletal muscle regeneration. The results confirmed that ERα plays a crucial protective role in myocyte differentiation and skeletal muscle regeneration in *mdx* mice, which is the innovative aspect of this study. However, other cell types also exhibit significant biological activities during the progression of DMD in *mdx* mice. Therefore, the role of ERα in other cell types or tissues in the context of DMD cannot be ruled out. Future research will investigate the expression levels and biological functions of ERα in different cell types within the muscle tissues of *mdx* mice or DMD patients.

Chronic inflammation, oxidative stress and muscle fibre necrosis are the primary pathology of DMD [3]. Glucocorticoids have been used to treat DMD since 1980 because they possess anti‐inflammatory properties and prolong patient mobility. However, long‐term use of glucocorticoids leads to various side effects, including weight gain and osteoporosis [34]. Therefore, glucocorticoids are not the most ideal therapeutic option. Gene therapy is currently the most recommended treatment for DMD [35]. In recent years, gene therapies such as stop codon readthrough, exon skipping and micro‐dystrophin replacement have been applied in the clinical treatment of DMD [S6–S8]. The advantage of gene therapy lies in its ability to restore or partially restore the expression of functional dystrophin. However, currently approved gene therapies do not completely address or cure all DMD patients, and the level of dystrophin protein expression remains relatively low. Consequently, the development of small‐molecule drugs targeting downstream on skeletal muscle inflammation, fibrosis and functional degradation is also essential [6]. Thus, the search for drugs to treat DMD still needs to be constantly explored. In this study, we discovered that E2 can promote myoblast differentiation and improve skeletal muscle regeneration in *mdx* mice by activating ERα in myocyte. Therefore, targeting myocyte ERα to promote skeletal muscle regeneration may represent a novel therapeutic strategy for DMD.

Muscle regeneration from satellite cells is regulated by the myogenic regulatory factor family. Among these factors, MyoD protein is expressed in activated satellite cells and their progeny (i.e., myoblasts, myocytes and myotubes) [S9, S10]. During development, MyoD, MyoG and MyHC are considered a key determinant of myogenesis [36]. Our results demonstrated that E2 activated ERα in myocyte to enhance MyoD‐mediated myoblast differentiation and muscle regeneration. Compared to *mdx* mice, ERα^mKO^ *mdx* mice exhibit exacerbated pathology characterized by increased inflammation, fibrosis and impaired skeletal muscle regeneration. E2 demonstrated therapeutic benefits for skeletal muscle regeneration in *mdx* mice, which were abolished in ERα^mKO^ *mdx* mice. These results highlight that the therapeutic efficacy of E2 relied on its interaction with ERα in myocyte.

Bioinformatics analysis and clinical sample experiments revealed upregulation of ERα in both DMD patients and *mdx* mice muscles. Consequently, our initial approach involved considering that oestrogen receptor antagonists to downregulate ERα could yield therapeutic benefits. Interestingly, FVT only reduced CK levels and alleviation of inflammation in *mdx* mice muscles. FVT failed to ameliorate muscle fibre loss and skeletal muscle regeneration disorders. We speculated that the microenvironment of skeletal muscle tissue in *mdx* mice is complex and likely harbouring various cell types in addition to myocytes. Furthermore, FVT acts as a broad‐spectrum oestrogen receptor antagonist and might impede oestrogen signalling in inflammatory cells, modulating immune cell function and influencing the production of inflammatory mediators [30]. Nonetheless, the current understanding of the effects of FVT on inflammation remains limited, needed for further research to corroborate its therapeutic efficacy.

ERRα is an orphan nuclear receptor that plays a crucial role in regulating various aspects of mitochondrial biogenesis and oxidative metabolism [37]. The overexpression of ERRα in mouse muscle enhances mitochondrial oxidative metabolism and induces the transition of muscle fibres to Type I oxidative fibres [38]. Muscle‐specific knockout of ERRα in mice leads to regeneration disorders following skeletal muscle injury [24]. However, studies have demonstrated high sequence homology between ERα and ERRα and evidence of ERα‐ERRα transcriptional cross‐talk in breast cancer tissues [39]. Research on the specific mechanisms of ERRα in DMD remains limited. In our study, we observed that knocking down ERα simultaneously inhibited ERRα expression, whereas overexpressing ERRα did not affect ERα. This suggested that ERRα is downstream of ERα and is regulated by it. In experiments with *mdx* mice and in vitro cell cultures, the increased *Myod1* following ERα activation was attributed to ERRα's promotion of MyoD transcription. ERα in myocyte played a protective role in skeletal muscle regeneration in *mdx* mice. E2 activated the biological function of ERα, thereby activating the ERα/ERRα/MyoD signalling pathway to promote myogenesis and improve the pathological condition in *mdx* mice. E2 regulated the transcription factor ERRα through ERα in myocyte, enhancing the transcriptional activation of MyoD to promote myocyte differentiation. ERRα is known to be a critical functional partner of PGC‐1α in skeletal muscle, forming functional dimers that act as transcriptional activators [40]. In this study, we found *mdx* mice have lower levels of PGC‐1α and ERRα, which is consistent with published reports [S11] and our supplementary clinical data (Figure S6D). ERα in myocyte played a protective role in skeletal muscle regeneration in *mdx* mice. Reportedly, in DMD patients and *mdx* mice, progressive skeletal muscle necrosis and atrophy suppressed the PGC‐1α/ERRα signalling pathway, leading to a reduced mitochondrial metabolic capacity in skeletal muscle tissue [S12]. Here, we demonstrated ERα‐PGC‐1α/ERRα‐MyoD pathway could play a protective role in DMD. However, there is a lack of enough ERα agonist to exert its biological function including the activation of PGC‐1α/ERRα pathway, although ERα overexpressed in DMD pathological process which might be happened as a compensatory effect. Thus, exogenous stimulant (like E2) could utilize overexpressed ERα (in *mdx* mice but not in ERα^mKO^ *mdx* mice) to activate PGC‐1α/ERRα‐induced skeletal muscle regeneration, improving pathological symptoms of DMD.

DMD predominantly affects males, but both male and female muscles are susceptible to muscular dystrophy, with some studies indicating differences between them [S13]. These differences are mainly attributed to variations in energy metabolism, fibre type composition and contraction speed, which are largely influenced by oestrogen levels in females [S14]. Previous research has shown that oestrogen receptor expression can vary across different muscle types [S15]. The observed upregulation of ERα in the EDL muscle may be linked to metabolic disturbances in skeletal muscles under the pathological conditions present in *mdx* mice. A related study focused on homozygous *mdx* female mice to explore the effects of oestrogen on muscle strength and recovery after injury. The results indicated that oestrogen deficiency impairs the recovery of muscle strength following skeletal muscle injury and oestradiol supplementation could promote skeletal, metabolic and mitochondrial function [25]. Further research is warranted to investigate whether ERα contributes to sex differences in the *mdx* mouse phenotype.

In conclusion, we confirmed the upregulation of ERα in both DMD patients and *mdx* mice muscles, with ERα in myocyte playing a protective role in skeletal muscle regeneration. E2 was found to activate the biological functions of ERα in myocyte, upregulating ERα and ERRα to facilitate the transcriptional regulation of the myogenic factor MyoD, thereby enhancing myoblast differentiation, improving the loss of muscle fibres in *mdx* mice and promoting skeletal muscle regeneration.

## Author Contributions

X.H. and S.L.: validation, investigation, visualization, methodology, writing – original draft and editing. H.W., W.H., H.T. and G.G.: methodology, validation and investigation. S.F. and D.X.: conceptualization. L.Z and X.L.: resources. L.Z. and Z.J.: conceptualization, formal analysis, funding acquisition, editing. Q.Y.: conceptualization, supervision, funding acquisition, project administration, writing – original and editing.

## Conflicts of Interest

The authors declare no conflicts of interest.

## Supporting information


**Table S1** The sequence of primers (mouse).
**Figure S1.** Biochemical and histological analysis of DMD patient and *mdx* mice. (A) IF staining of CD68 in DMD patient and healthy control. Scale bar: 25 μm. (B) IF staining of dystrophin in mouse skeletal muscle. Scale bar: 80 μm. (C) The levels of CK and LDH in mouse serum. (D) Behavioural functions in mice. (E) Relative weight of GAS and TA of mice. (F) Quantification of central nucleated muscle fibres in GAS, TA and DIA of mice. (G) Quantification of cross‐sectional area of GAS, TA and DIA of mice. (H) IF staining of MyHC and laminin and quantification of MyHC expression in mouse muscle. Scale bar: 100 μm. (I) IF staining of eMyHC and laminin and quantification of eMyHC expression in mouse muscle. Scale bar: 100 μm. (J) IF staining of CD68 and laminin and quantification of CD68 expression in mouse muscle. Scale bar: 100 μm. (K) IF staining of MyHC I and MyHC IIA and quantification expression in GAS. Scale bar: 100 μm. (L) IF staining of eMyHC and ERα in GAS of mice. Scale bar: 50 μm. (M) Masson staining of mouse muscle. Scale bar: 50 μm. Eight‐ to ten‐week‐old males were used (*n* = 5–6). The data are presented as the means ± SDs; **p < 0.01, ***p < 0.001.
**Figure S2.** Biochemical and histological analysis of *mdx* mice with FVT treatment. (A) Changes in the body weight of mice 4 weeks after subcutaneous FVT injection. (B) Relative weight of GAS and TA of mice. (C) Quantification of central nucleated muscle fibres in GAS, TA and DIA of mice. (D) Quantification of cross‐sectional area of GAS, TA and DIA of mice. (E) IF staining of MyHC and laminin, and quantification of MyHC expression in mouse muscle. Scale bar: 100 μm. (F) IF staining of CD68 and laminin and quantification of CD68 expression in mouse muscle. Scale bar: 100 μm. G. Masson staining of mouse muscle after treatment with FVT. Scale bar: 50 μm. Eight‐ to ten‐week‐old males were used (*n* = 5). The data are presented as the means ± SDs; *p < 0.05, **p < 0.01, ***p < 0.001.
**Figure S3.** Biochemical and histological analysis of *mdx* mice with E2 treatment. (A) Changes in the body weight of mice 4 weeks after subcutaneous injection of E2. (B) Relative weight of GAS and TA of mice. (C) Quantification of central nucleated muscle fibres in GAS, TA and DIA of mice. (D) Quantification of cross‐sectional area of GAS, TA and DIA of mice. (E) IF staining of MyHC and laminin, and quantification of MyHC expression in mouse muscle. Scale bar: 100 μm. (F) IF staining of CD68 and laminin and quantification of CD68 expression in mouse muscle. Scale bar: 100 μm. (G) Masson staining of mouse muscle after treatment with FVT. Scale bar: 50 μm. (H) IF staining of ERα, CD68 and eMyHC in the GASs of *mdx* mice. Scale bar: 50 μm. (I) H&E staining of WT group mouse muscle after treatment with FVT or E2. Scale bar: 50 μm. Eight‐ to ten‐week‐old males were used (*n* = 5). The data are presented as the means ± SDs; *p < 0.05, **p < 0.01, ***p < 0.001.
**Figure S4.** Transfection efficiency of si‐*Esr1*. (A) Protein expression of ERα on *mdx* primary myoblast cells after the administration of E2 and FVT. (B) *Esr1* mRNA expression in C2C12 cells after the transfection of the *si‐Esr1* sequence. (C) IF staining of MyHC and ERα on C2C12 cells after the transfection of si‐*Esr1* and quantification of the length of myotubes. (D) Protein expression of ERα, MyoG and MyoD in C2C12 cells after the transfection of the si‐*Esr1* sequence. IF staining scale bar: 100 μm. The data are presented as the means ± SDs; **p < 0.01, ***p < 0.001.
**Figure S5.** Biochemical and histological analysis of ERα^mKO^ *mdx* mice with E2 treatment. (A) Protein expression of ERα in the GAS, TA and DIA muscles of *mdx* and ERα^mKO^ *mdx* mice. (B) Relative weight of GAS and TA muscles of *mdx* and ERα^mKO^ *mdx* mice. (C) Quantification of central nucleated muscle fibres in GAS, TA and DIA muscles of *mdx* and ERα^mKO^ *mdx* mice. (D) Masson staining of the muscle of *mdx* mice, ERα^mKO^ *mdx* mice and ERα^mKO^ *mdx* mice after treatment with E2. Scale bar: 50 μm. (E) Quantification of cross‐sectional area of GAS, TA and DIA of mice. (F) IF staining of MyHC and laminin and quantification of MyHC expression in muscles of *mdx* and ERα^mKO^ *mdx* mice. Scale bar: 100 μm. (G) IF staining of CD68 and laminin and quantification of CD68 expression in muscles of *mdx* and ERα^mKO^ *mdx* mice. Scale bar: 100 μm. (H) IF staining of MyHC I and MyHC IIA and quantification of MyHC I and MyHC IIA expression in GAS of WT mice, *mdx* mice, ERα^mKO^ *mdx* mice and ERα^mKO^ mice after treatment with E2. IF staining scale bar: 100 μm. Eight‐ to ten‐week‐old males were used (*n* = 5). The data are presented as the means ± SDs; **p < 0.01, ***p < 0.001.
**Figure S6.** Transfection efficiency of OE‐*Esrra*. (A) *Esrra* mRNA expression in C2C12 cells after the transfection of the *Esrra* overexpression plasmid. (B) Protein expression of ERRα and ERα in C2C12 cells after the administration of OE‐*Esrra*. (C) Comparison of *ESRRA* expression between DMD patients and healthy controls in the GSE1004_GLP8300 dataset. (D) Comparison of *ESR1*, *ESRRA* and *PPARGC1A* expression between DMD patients and healthy controls in the GSE38417 dataset. The data are presented as the means ± SDs; **p < 0.01.
**Figure S7.** Transfection efficiency of si‐*Esrra*. (A) *Esrra* mRNA expression in C2C12 cells after the transfection of the si‐*Esrra*. (B,C) Protein expression of ERR, PGC‐1α, ERα, MyoD and MyoG on C2C12 cells after the administration of si‐*Esrrα* and E2. (D) mRNA expression of *Myod1* on C2C12 cells after the administration of si‐*Esrra* and E2. (E) IF staining of MyHC on C2C12 cells after the transfection of the administration of si‐*Esrra* and E2 and fusion index calculated as the average number of nuclei in MyHC‐positive C2C12 cells. (F) CHIP assay on C2C12 cell and C2C12 cells treated with E2. Recruiting of ERRα to MyoD promoter binding site. IF staining scale bar: 100 μm. The data are presented as the means ± SDs; *p < 0.05, **p < 0.01.

**Data S1** Supporting Information.
