# Supplementary material for: Oestrogen Receptor Alpha in Myocyte Maintains Muscle Regeneration in Duchenne Muscular Dystrophy
Source: J Cachexia Sarcopenia Muscle. 2025 Apr 21;16(2):e13807. doi: 10.1002/jcsm.13807 (PMC12011492; doi:10.1002/jcsm.13807)
Supplement: Supplementary file 1 — Table S1 The sequence of primers (mouse). Figure S1. Biochemical and histological analysis of DMD patient and mdx mice. (A) IF staining of CD68 in DMD patient and healthy control. Scale bar: 25 μm. (B) IF staining of dystrophin in mouse skeletal muscle. Scale bar: 80 μm. (C) The levels of CK and LDH in mouse serum. (D) Behavioural functions in mice. (E) Relative weight of GAS and TA of mice. (F) Quantification of central nucleated muscle fibres in GAS, TA and DIA of mice. (G) Quantification of cross‐sectional area of GAS, TA and DIA of mice. (H) IF staining of MyHC and laminin and quantification of MyHC expression in mouse muscle. Scale bar: 100 μm. (I) IF staining of eMyHC and laminin and quantification of eMyHC expression in mouse muscle. Scale bar: 100 μm. (J) IF staining of CD68 and laminin and quantification of CD68 expression in mouse muscle. Scale bar: 100 μm. (K) IF staining of MyHC I and MyHC IIA and quantification expression in GAS. Scale bar: 100 μm. (L) IF staining of eMyHC and ERα in GAS of mice. Scale bar: 50 μm. (M) Masson staining of mouse muscle. Scale bar: 50 μm. Eight‐ to ten‐week‐old males were used (n = 5–6). The data are presented as the means ± SDs; **p < 0.01, ***p < 0.001. Figure S2. Biochemical and histological analysis of mdx mice with FVT treatment. (A) Changes in the body weight of mice 4 weeks after subcutaneous FVT injection. (B) Relative weight of GAS and TA of mice. (C) Quantification of central nucleated muscle fibres in GAS, TA and DIA of mice. (D) Quantification of cross‐sectional area of GAS, TA and DIA of mice. (E) IF staining of MyHC and laminin, and quantification of MyHC expression in mouse muscle. Scale bar: 100 μm. (F) IF staining of CD68 and laminin and quantification of CD68 expression in mouse muscle. Scale bar: 100 μm. G. Masson staining of mouse muscle after treatment with FVT. Scale bar: 50 μm. Eight‐ to ten‐week‐old males were used (n = 5). The data are presented as the means ± SDs; *p < 0.05, **p < 0.01, ***p < 0.00 [file JCSM-16-e13807-s002.docx]

**Oestrogen receptor alpha in myocyte maintains muscle regeneration in Duchenne muscular dystrophy**

Xiaofei Huang^1,6^, Sijia Li^1,6^, Huna Wang^1^, Lei Zhao^2^, Xihua Li^2^, Shusheng Fan^1^, Wanting Hu^1^, Haowei Tong^1^, Guangyao Guo^1^, Dengqiu Xu^3^, Luyong Zhang^1,4^, Zhenzhou Jiang^1,5,7*^, Qinwei Yu^1*^

^1^New Drug Screening and Pharmacodynamics Evaluation Center, China Pharmaceutical University, Nanjing, China

^2^Department of Neurology, Children's Hospital of Fudan University, Shanghai, China

^3^Department of Hepatobiliary Surgery, Innovative Institute of Tumor Immunity and Medicine (ITIM), Anhui Province Key Laboratory of Tumor Immune Microenvironment and Immunotherapy, The First Affiliated Hospital of Anhui Medical University, Hefei, China.

^4^Center for Drug Research and Development, Guangdong Pharmaceutical University, Guangzhou, China

^5^Key Laboratory of Drug Quality Control and Pharmacovigilance, Ministry of Education, China Pharmaceutical University, Nanjing, China

^6^These authors contributed equally.

^7^Lead contact.

*Correspondence: No. 24, Tongjia Lane, Gulou District, Nanjing 210009, China.

Email: beaglejiang@cpu.edu.cn (Zhenzhou Jiang), yuqinwei7213@cpu.edu.cn (Qinwei Yu)

1. **Supplement Methods**
   1. **Grip strength testing**

The muscle grip test is used to measure the grip strength of mice and to evaluate muscle strength and skeletal muscle contraction. Grip strength was assessed using a grip strength meter (YSL-13A, Jinan Yiyan Technology Development Co. Ltd., Jinan China). Briefly, the mouse was allowed to grasp the shafts on the holding board with its four limbs and was gently pulled back while holding its tail at a constant speed. Then, the maximum grip strength was recorded. Each mouse was subjected to six trials per session, with a 30-sec recovery period between trials.

- 1. **Hanging test**

The hanging test was performed as described previously. Basically, a 55 cm wide and 2 mm thick metallic wire was secured to two vertical stands. The wire was maintained 35 cm above a layer of bedding material. The mouse was allowed to grasp the middle of the wire on all four limbs. Then, the tail was released, and the time until the mouse completely relaxed its grasp and fell down was recorded. Each mouse was subjected to three trials per session, and 30 sec of recovery was evaluated by using the following equation: Holding Impulse (s*g) = Body mass (grams) × Hanging Time (sec) as an outcome measure.

- 1. **qRT-PCR**

The total RNA was isolated from cell with RNA isolater Total RNA Extraction Reagent (Vavyme Cat# R401). And transcribing RNA to cDNA with HiScript II Q RT SuperMix for qPCR (Vazyme Cat# R223). qRT-PCR was performed via the StepOne Plus TM qRT-PCR system (Life Technologies, USA) by using AceQ qRT-PCR SYBR Green Master Mix (Vazyme Cat# Q141) with custom made plates. The mRNA expression of genes was normalized to the mean of Gapdh and quantified by the 2-ΔΔct method. Primers were purchased from Invitrogen with the following **Table 1**.

**Table 1**

The sequence of primers (mouse)

| Gene | Forward primer | Reverse primer |
| --- | --- | --- |
| *Esr1* | TCTGCCAAGGAGACTCGCTACT | GGTGCATTGGTTTGTAGCTGGAC |
| *Esrra* | ACTACGGTGTGGCATCCTGTGA | GGTGATCTCACACTCATTGGAGG |
| *Myod1* | CCACTCCGGGACATAGACTTG | AAAAGCGCAGGTCTGGTGAG |
| *Gapdh* | TGACCACAGTCCATGCCATCACTG | ATGACCTTGCCCACAGCCTTGG |

- 1. **Serum biochemical analysis**

Serum creatine kinase (CK) and lactic dehydrogenase (LDH) were evaluated as measurements of whole-body muscle damage. Blood samples were collected and incubated at room temperature for 30 min. Then, the samples were centrifuged at 4,000 rpm for 10 min at 4°C to obtain serum. The serum was sent to Jiangsu Integrated Traditional Chinese and Western Medicine Hospital, and Nanjing Tech University for the detection of CK and LDH.

- 1. **Muscle fiber measurement, nuclear centralization, and fiber type statistics**

To analyze muscle fibers, first set the scale for each image by selecting “Analysis” > “Set Scale” in FIJI. Then, use the “Freehand selections” tool to trace the contours of individual muscle fibers. After outlining the muscle fibers, navigate to “Analyze” > “Measure” to calculate the area of the selected region. Repeat this process for all muscle fibers within the image. The “ROI Manager” tool can be used to manage and label multiple regions for efficient measurement. The CSA of muscle fibers was grouped in ascending order with intervals of 500 μm. The cumulative percentage for each group was calculated, and a curve plot was generated.

For the quantification of nuclear centralization, begin by selecting “Plugins” > “Analyze” > “Cell Counter” to count the total number of muscle fibers and muscle fiber with centralized nuclei in the image. Utilize the “ROI Manager” tool to label and categorize muscle fibers based on central nuclei. The percentage of fibers with centralized nuclei was calculated and represented using a violin plot. For the quantification of muscle fiber type , begin by selecting “Plugins” > “Analyze” > “Cell Counter” to count the total number of muscle fibers and the different type fibers identified by the different IF staining color in the image. Utilize the “ROI Manager” tool to label and categorize muscle fibers based on muscle fiber type. The proportions of different muscle fiber types were calculated and depicted as a bar graph.

- 1. **CHIP assay**

CHIP from C2C12 cells was administrated with E2. C2C12 cells were crosslinked with 1% formaldehyde for 10 minutes at room temperature, followed by quenching with 125 mM glycine for 5 minutes. The cells were then harvested and lysed in ice-cold lysis buffer (1% SDS, 1 mM PMSF) supplemented with protease inhibitors. The chromatin was sheared into approximately 200-800 bp fragments using Ultrasonic disintegrator. Immunoprecipitation was performed overnight at 4°C using ERRα antibodies against the target protein. As a negative control, a non-specific IgG antibody was used. The antibody-chromatin complexes were captured with protein A/G-agarose beads (Santa Cruz Biotechnology), washed sequentially with low-salt, high-salt, and LiCl buffers, and then eluted in 1% SDS, 0.1 M NaHCO3. The crosslinks were reversed by incubation at 65°C overnight, and purified DNA. Quantitative PCR (qPCR) was used to analyze the enrichment of specific DNA sequences, normalized to input DNA. The relative binding of the protein to the DNA was calculated using the 2^(-ΔΔCt) method.

- 1. **Public data analysis**

Reanalyzed the public data from GSE1004_GLP8300 in the GEO database. This dataset includes DNA microarray data from quadriceps muscle biopsy specimens of 12 male DMD patients (aged 1-8 years) and 11 healthy controls (including 7 young males, 2 adult males, 1 young female, and 1 adult female, from the USA). We reanalyzed and compared the *ESR1* levels between the healthy controls and DMD patients. The estradiol dataset used in the study was obtained from the Therapeutic Target Database (D08QMX) and PubChem Database (5757), focusing on the biological targets that estradiol may affect. The ERα dataset was sourced from the NCBI-PubChem database (P19785), targeting the biological targets potentially interacting with ERα. The DMD dataset was obtained from the MalaCards database (MSC157), concentrating on the biological targets altered during the progression of DMD. By intersecting these three datasets, 62 differentially expressed genes were identified. Subsequently, GO enrichment analysis and KEGG pathway enrichment analysis were performed on the 62 differentially expressed genes using the Metascape online tool. The expression heatmap and volcano plot distributions of these 62 differentially expressed genes were analyzed in the GSE1004_GLP8300 dataset. Through protein-protein interaction (PPI) network analysis, we selected ERRα as the target gene most strongly correlated with *Esr1* and *Myod1* among the 12 significantly altered DEGs.

1. **Supplement References**

1. Woo M, Tanabe Y, Ishii H, Nonaka I, Yokoyama M, Esaki K. Muscle fiber growth and necrosis in dystrophic muscles: a comparative study between dy and mdx mice. J Neurol Sci. 1987;82:111-22.

2. Yucel N, Chang AC, Day JW, Rosenthal N, Blau HM. Humanizing the mdx mouse model of DMD: the long and the short of it. NPJ Regen Med. 2018;3:4.

3. Yasar P, Ayaz G, User SD, Gupur G, Muyan M. Molecular mechanism of estrogen-estrogen receptor signaling. Reprod Med Biol. 2017;16:4-20.

4. Szklarczyk D, Kirsch R, Koutrouli M, Nastou K, Mehryary F, Hachilif R, et al. The STRING database in 2023: protein-protein association networks and functional enrichment analyses for any sequenced genome of interest. Nucleic Acids Res. 2023;51:D638-D46.

5. Paterni I, Granchi C, Katzenellenbogen JA, Minutolo F. Estrogen receptors alpha (ERalpha) and beta (ERbeta): subtype-selective ligands and clinical potential. Steroids. 2014;90:13-29.

6. McDonald CM, Campbell C, Torricelli RE, Finkel RS, Flanigan KM, Goemans N, et al. Ataluren in patients with nonsense mutation Duchenne muscular dystrophy (ACT DMD): a multicentre, randomised, double-blind, placebo-controlled, phase 3 trial. Lancet. 2017;390:1489-98.

7. Syed YY. Eteplirsen: First Global Approval. Drugs. 2016;76:1699-704.

8. Willcocks RJ, Forbes SC, Walter GA, Sweeney L, Rodino-Klapac LR, Mendell JR, et al. Assessment of rAAVrh.74.MHCK7.micro-dystrophin Gene Therapy Using Magnetic Resonance Imaging in Children With Duchenne Muscular Dystrophy. JAMA Netw Open. 2021;4:e2031851.

9. Conerly ML, Yao Z, Zhong JW, Groudine M, Tapscott SJ. Distinct Activities of Myf5 and MyoD Indicate Separate Roles in Skeletal Muscle Lineage Specification and Differentiation. Dev Cell. 2016;36:375-85.

10. Yagi M, Ji F, Charlton J, Cristea S, Messemer K, Horwitz N, et al. Dissecting dual roles of MyoD during lineage conversion to mature myocytes and myogenic stem cells. Genes Dev. 2021;35:1209-28.

11. Suntar I, Sureda A, Belwal T, Sanches Silva A, Vacca RA, Tewari D, et al. Natural products, PGC-1 alpha , and Duchenne muscular dystrophy. Acta Pharm Sin B. 2020;10:734-45.

12. Summermatter S, Santos G, Perez-Schindler J, Handschin C. Skeletal muscle PGC-1alpha controls whole-body lactate homeostasis through estrogen-related receptor alpha-dependent activation of LDH B and repression of LDH A. Proc Natl Acad Sci U S A. 2013;110:8738-43.

13. Hermes TA, Kido LA, Macedo AB, Mizobuti DS, Moraes LHR, Somazz MC, et al. Sex influences diaphragm muscle response in exercised mdx mice. Cell Biol Int. 2018;42:1611-21.

14. Frank DE, Schnell FJ, Akana C, El-Husayni SH, Desjardins CA, Morgan J, et al. Increased dystrophin production with golodirsen in patients with Duchenne muscular dystrophy. Neurology. 2020;94:e2270-e82.

15. Feder D, Rodrigues Barros Godoy I, Guimaraes Pereira ML, Silva CS, Nogueira Silvestre D, Fonseca FL, et al. Hormonal receptors in skeletal muscles of dystrophic mdx mice. Biomed Res Int. 2013;2013:604635.

1. **Supplement Figures**
   1. **Figure S1. Biochemical and histological analysis of DMD patient and *mdx* mice**


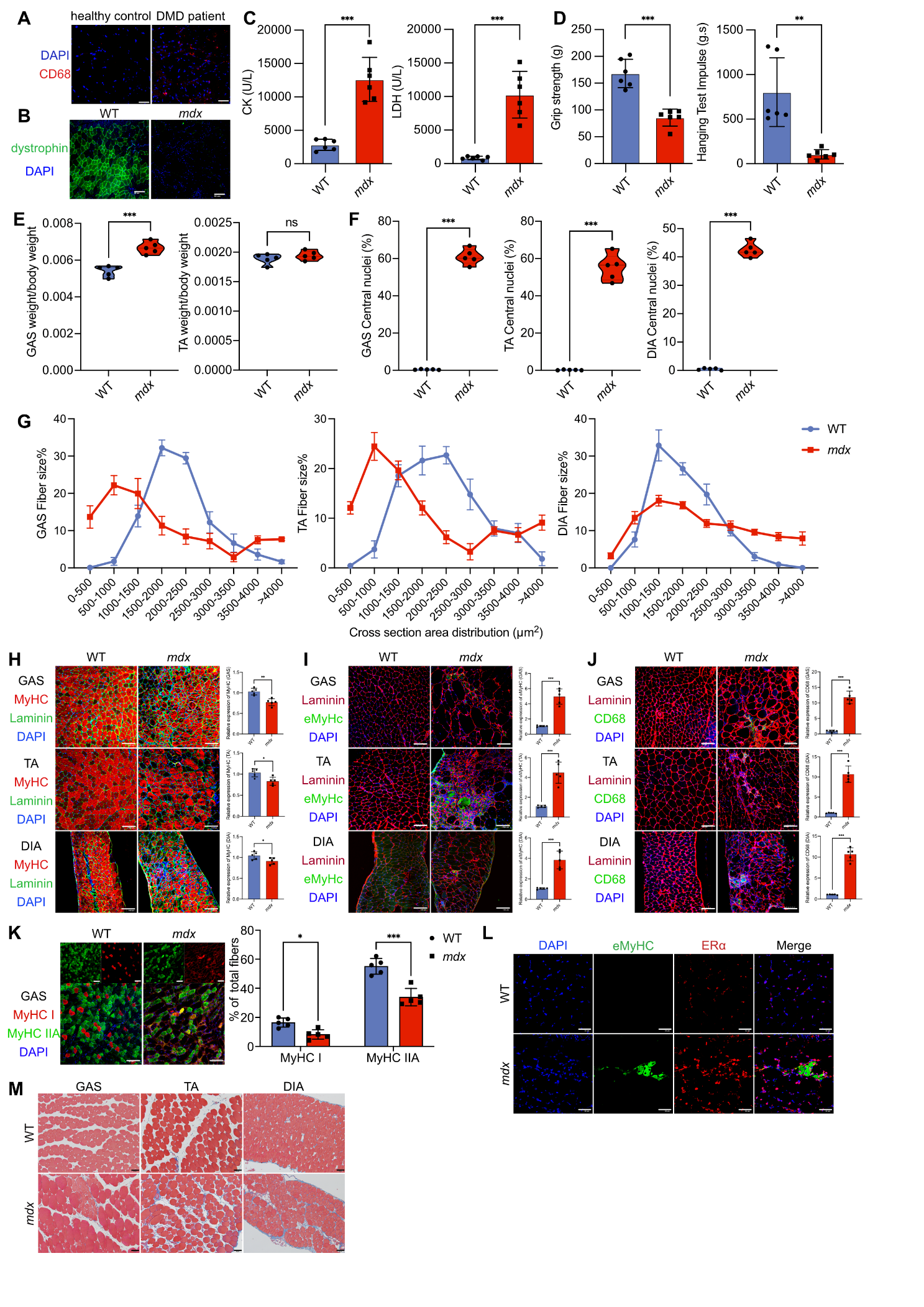


**Figure S1. Biochemical and histological analysis of DMD patient and *mdx* mice. A.** IF staining of CD68 in DMD patient and healthy control. Scale bar: 25 μm. **B.** IF staining of dystrophin in mouse skeletal muscle. Scale bar: 80 μm. **C.** The levels of CK and LDH in mouse serum. **D.** Behavioral functions in mice. **E.** Relative weight of GAS and TA of mice. **F.** Quantification of central nucleated muscle fibers in GAS, TA and DIA of mice. **G**. Quantification of cross-sectional area of GAS, TA and DIA of mice. **H**. IF staining of MyHC and laminin, and quantification of MyHC expression in mouse muscle. Scale bar: 100 μm. **I**. IF staining of eMyHC and laminin, and quantification of eMyHC expression in mouse muscle. Scale bar: 100 μm. **J.** IF staining of CD68 and laminin, and quantification of CD68 expression in mouse muscle. Scale bar: 100 μm. **K.** IF staining of MyHC I and MyHC IIA, and quantification expression in GAS. Scale bar: 100 μm. **L.** IF staining of eMyHC and ERα in GAS of mice. Scale bar: 50 μm. **M.** Masson staining of mouse muscle. Scale bar: 50 μm. Eight to ten-week-old males were used (n = 5-6). The data are presented as the means ± SDs; ***p* < 0.01, and ****p* < 0.001.

- 1. **Figure S2. Biochemical and histological analysis of *mdx* mice with FVT treatment.**


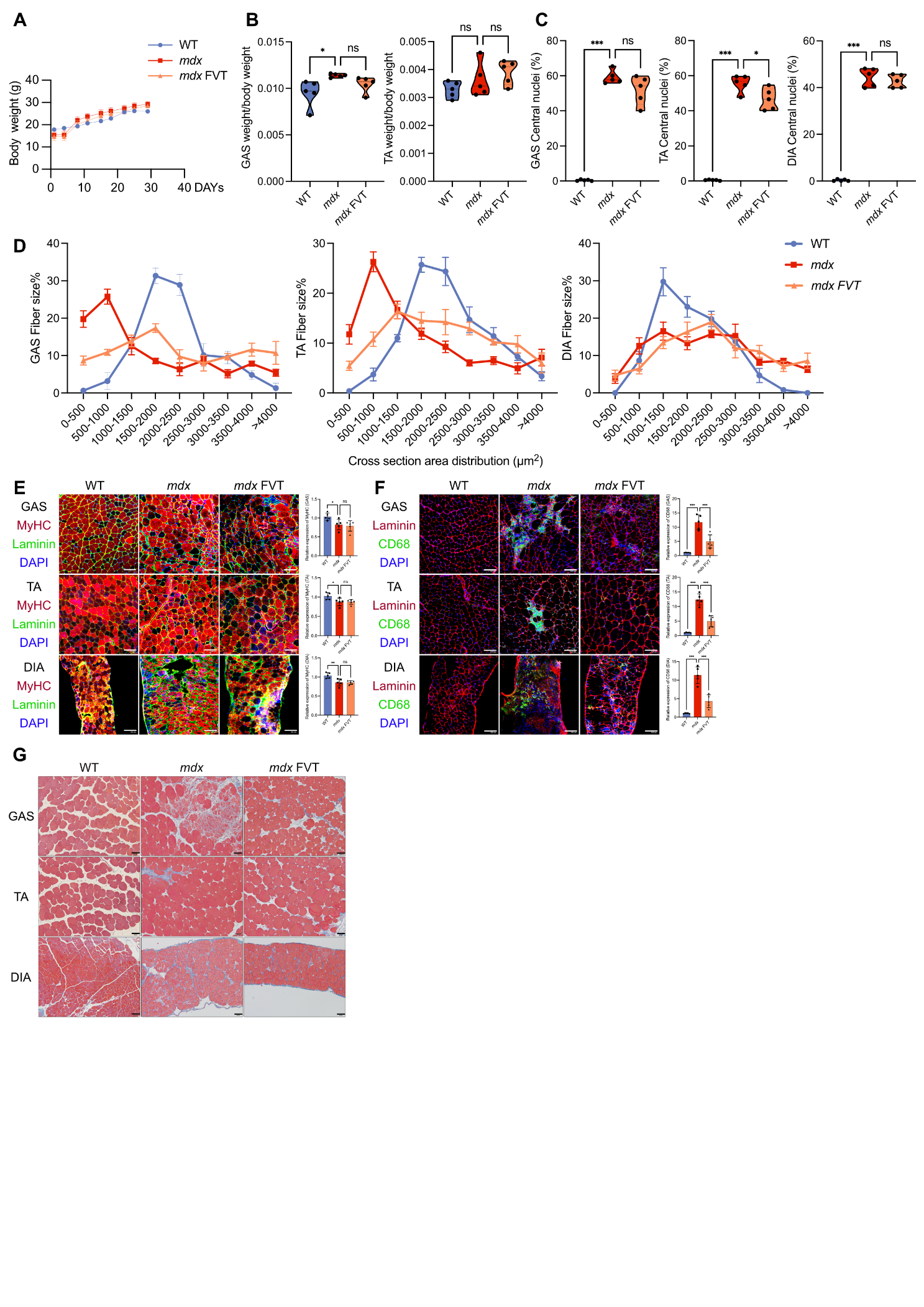


**Figure S2. Biochemical and histological analysis of *mdx* mice with FVT treatment. A**. Changes in the body weight of mice 4 weeks after subcutaneous FVT injection. **B**. Relative weight of GAS and TA of mice. **C**. Quantification of central nucleated muscle fibers in GAS, TA and DIA of mice. **D**. Quantification of cross-sectional area of GAS, TA and DIA of mice. **E.** IF staining of MyHC and laminin, and quantification of MyHC expression in mouse muscle. Scale bar: 100 μm. **F.** IF staining of CD68 and laminin, and quantification of CD68 expression in mouse muscle. Scale bar: 100 μm. **G.** Masson staining of mouse muscle after treatment with FVT. Scale bar: 50 μm. Eight- to ten-week-old males were used (n = 5). The data are presented as the means ± SDs; **p* < 0.05, ***p* < 0.01, ****p* < 0.001.

- 1. **Figure S3. Biochemical and histological analysis of *mdx* mice with E2 treatment.**


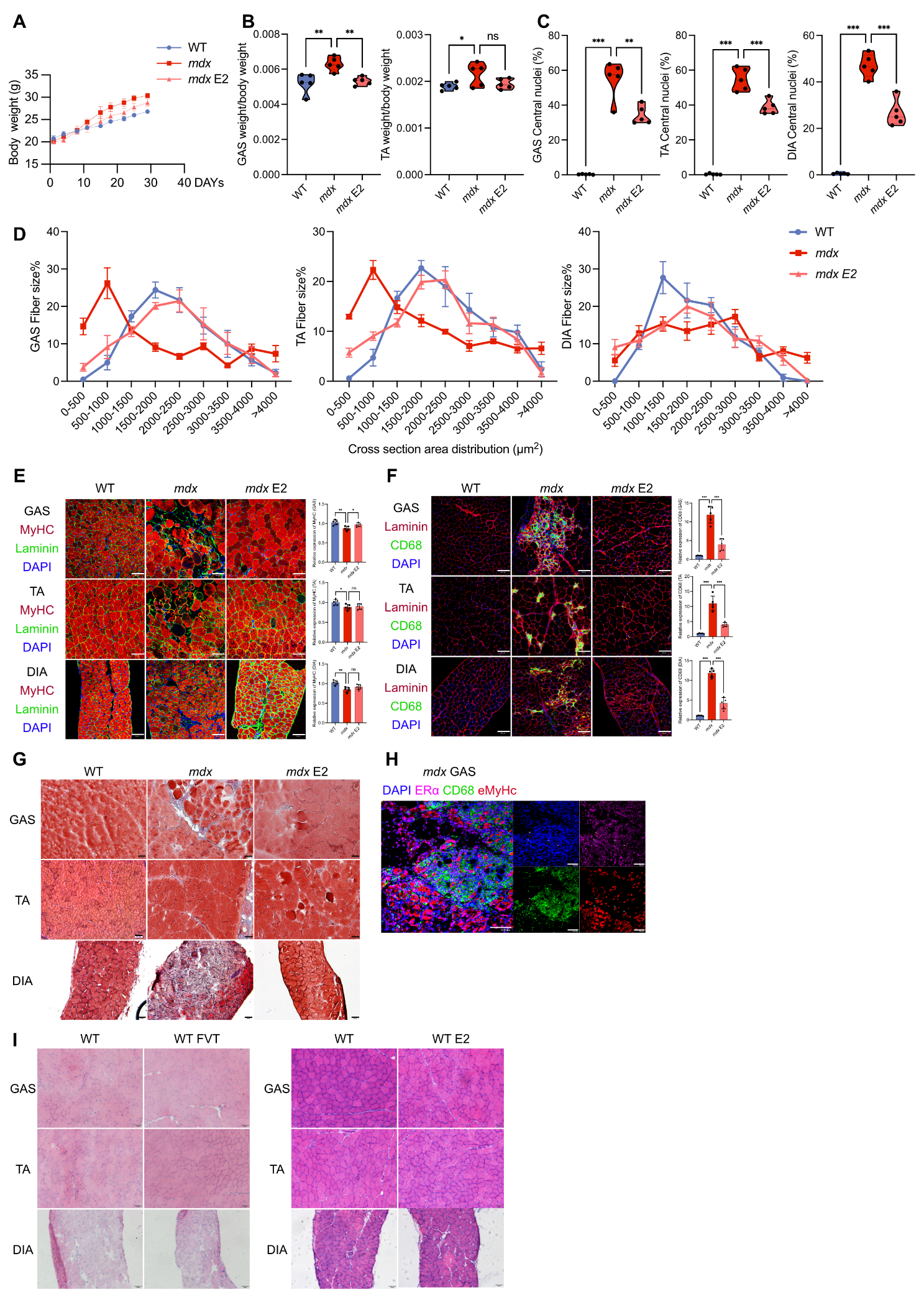


**Figure S3. Biochemical and histological analysis of *mdx* mice with E2 treatment. A**. Changes in the body weight of mice 4 weeks after subcutaneous injection of E2. **B**. Relative weight of GAS and TA of mice. **C**. Quantification of central nucleated muscle fibers in GAS, TA and DIA of mice. **D**. Quantification of cross-sectional area of GAS, TA and DIA of mice. **E.** IF staining of MyHC and laminin, and quantification of MyHC expression in mouse muscle. Scale bar: 100 μm. **F.** IF staining of CD68 and laminin, and quantification of CD68 expression in mouse muscle. Scale bar: 100 μm. **G.** Masson staining of mouse muscle after treatment with FVT. Scale bar: 50 μm. H. IF staining of ERα, CD68 and eMyHC in the GASs of *mdx* mice. Scale bar: 50 μm. I. H&E staining of WT group mouse muscle after treatment with FVT or E2. Scale bar: 50 μm. Eight- to ten-week-old males were used (n = 5). The data are presented as the means ± SDs; **p* < 0.05, ***p* < 0.01, ****p* < 0.001.

- 1. **Figure S4. Transfection efficiency of si-*Esr1*.**


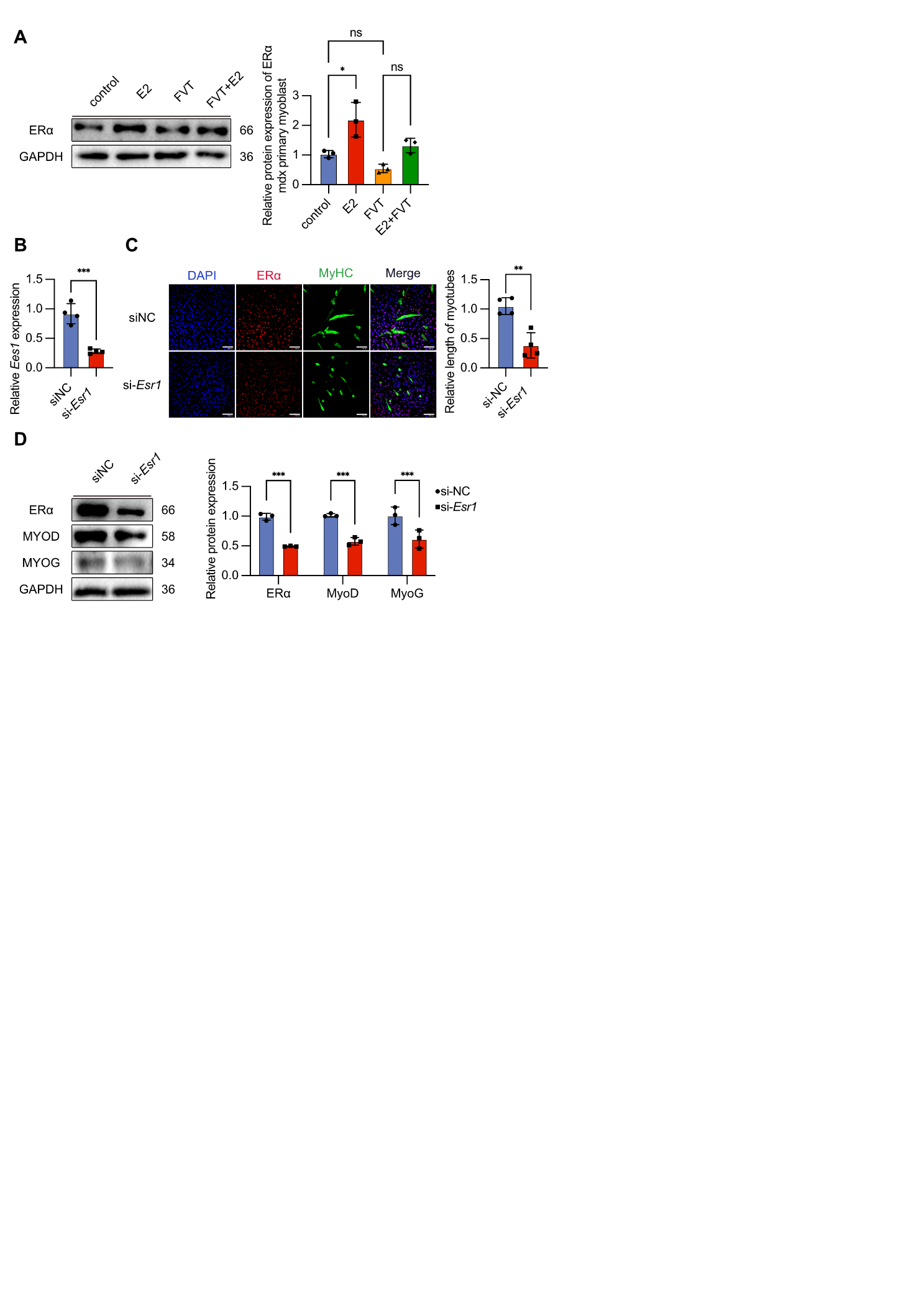


**Figure S4. Transfection efficiency of si-*Esr1*.** **A**. Protein expression of ERα on *mdx* primary myoblast cells after the administration of E2 and FVT. **B**. *Esr1* mRNA expression in C2C12 cells after the transfection of the *si-Esr1* sequence. **C**. IF staining of MyHC and ERα on C2C12 cells after the transfection of si-*Esr1* and quantification of the length of myotubes. D. Protein expression of ERα, MyoG and MyoD in C2C12 cells after the transfection of the si-*Esr1* sequence. IF staining scale bar: 100 μm. The data are presented as the means ± SDs; ***p* < 0.01, ****p* < 0.001.

- 1. **Figure S5. Biochemical and histological analysis of ERα^mKO^ *mdx* mice with E2 treatment.**


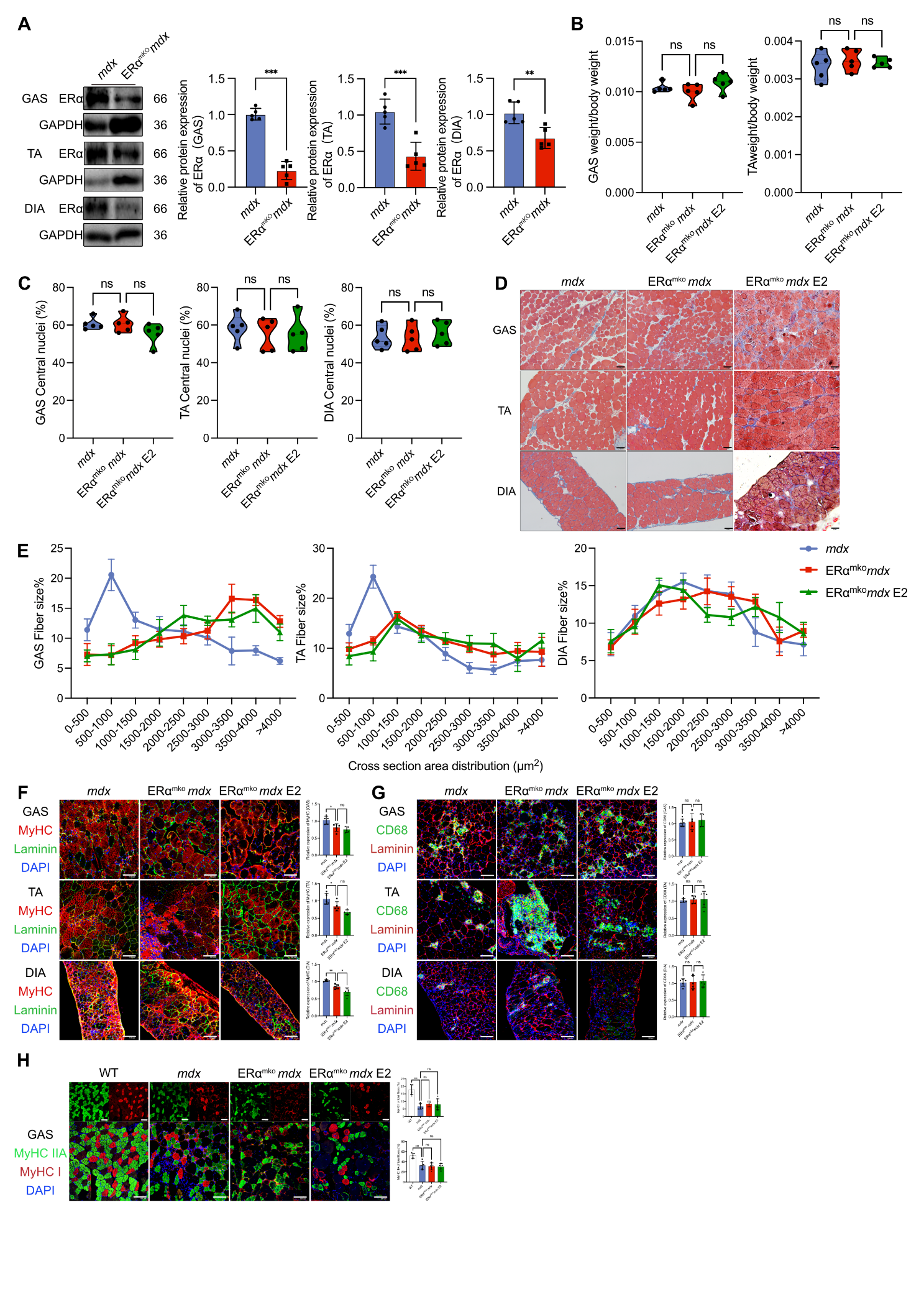


**Figure S5. Biochemical and histological analysis of ERα^mKO^ *mdx* mice with E2 treatment. A**. Protein expression of ERα in the GAS, TA and DIA muscles of *mdx* and ERα^mKO^ *mdx* mice. **B**. Relative weight of GAS and TA muscles of *mdx* and ERα^mKO^ *mdx* mice. **C**. Quantification of central nucleated muscle fibers in GAS, TA and DIA muscles of *mdx* and ERα^mKO^ *mdx* mice. **D**. Masson staining of the muscle of *mdx* mice, ERα^mKO^ *mdx* mice and ERα^mKO^ *mdx* mice after treatment with E2. Scale bar: 50 μm. **E**. Quantification of cross-sectional area of GAS, TA and DIA of mice. **F.** IF staining of MyHC and laminin, and quantification of MyHC expression in muscles of *mdx* and ERα^mKO^ *mdx* mice. Scale bar: 100 μm. **G.** IF staining of CD68 and laminin, and quantification of CD68 expression in muscles of *mdx* and ERα^mKO^ *mdx* mice. Scale bar: 100 μm. **H.** IF staining of MyHC I and MyHC IIA and quantification of MyHC I and MyHC IIA expression in GAS of WT mice, *mdx* mice, ERα^mKO^ *mdx* mice and ERα^mKO^ mice after treatment with E2. IF staining scale bar: 100 μm. Eight- to ten-week-old males were used (n = 5). The data are presented as the means ± SDs; ***p* < 0.01, ****p* < 0.001.

- 1. **Figure S6. Transfection efficiency of OE-*Esrra*.**

**
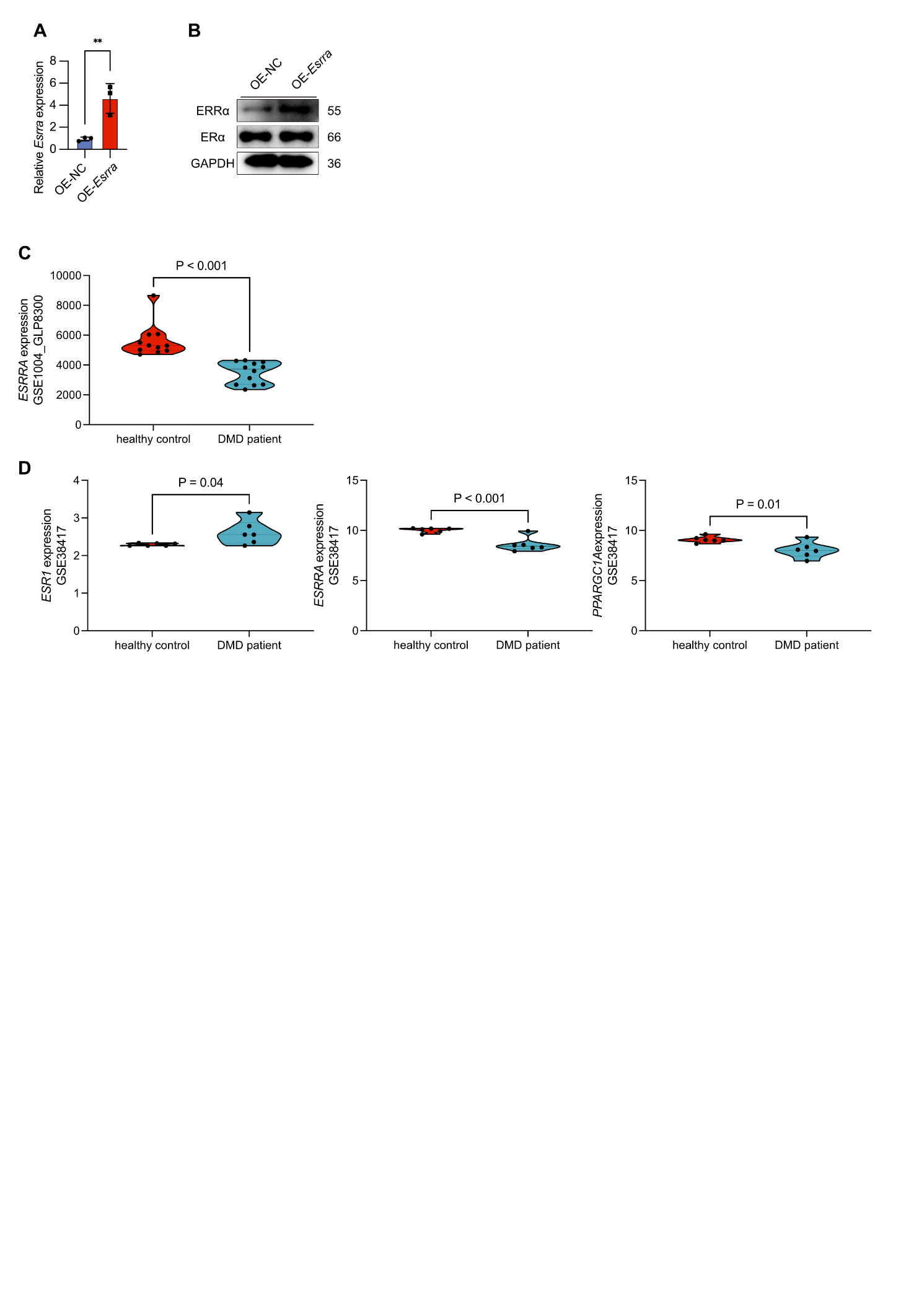
**

**Figure S6. Transfection efficiency of OE-*Esrra*.** **A**. *Esrra* mRNA expression in C2C12 cells after the transfection of the *Esrra* overexpression plasmid. **B**. Protein expression of ERRα and ERα in C2C12 cells after the administration of OE-*Esrra*. **C**. Comparison of *ESRRA* expression between DMD patients and healthy controls in the GSE1004_GLP8300 dataset. **D**. Comparison of *ESR1*, *ESRRA* and *PPARGC1A* expression between DMD patients and healthy controls in the GSE38417 dataset. The data are presented as the means ± SDs; ***p* < 0.01.

- 1. **Figure S7. Transfection efficiency of si-*Esrra*.**


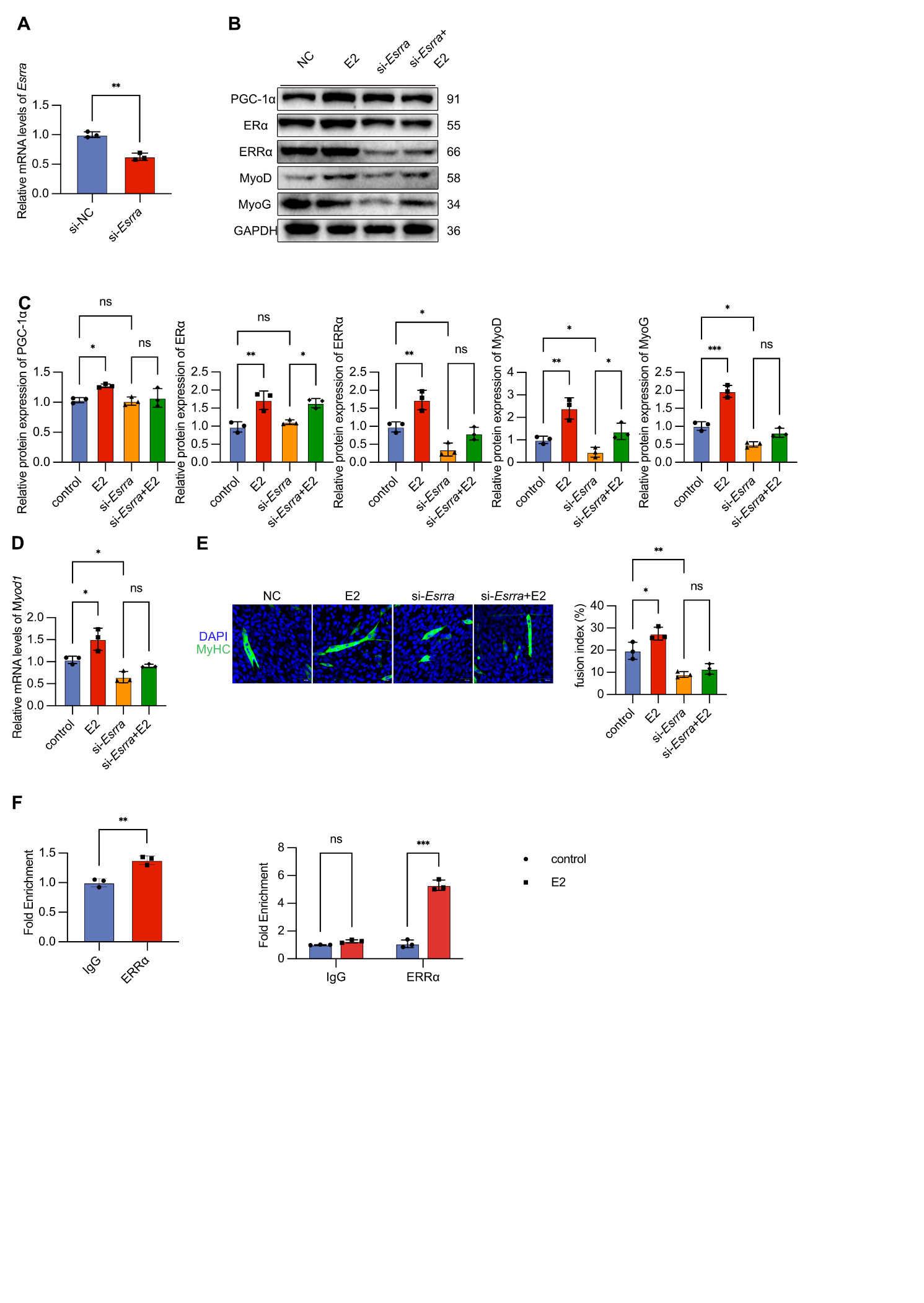


**Figure S7. Transfection efficiency of si-*Esrra*.** A. *Esrra* mRNA expression in C2C12 cells after the transfection of the si-*Esrra*. B and C. Protein expression of ERR, PGC-1α, ERα, MyoD and MyoG on C2C12 cells after the administration of si-*Esrrα* and E2. D. mRNA expression of *Myod1* on C2C12 cells after the administration of si-*Esrra* and E2. E. IF staining of MyHC on C2C12 cells after the transfection of the administration of si-*Esrra* and E2, and fusion index calculated as the average number of nuclei in MyHC positive C2C12 cells. F. CHIP assay on C2C12 cell, and C2C12 cells treated with E2. Recruiting of ERRα to MyoD promoter binding site. IF staining scale bar: 100 μm. The data are presented as the means ± SDs; **p* < 0.05, ***p* < 0.01.
