# Supplementary material for: Oestrogen Receptor Alpha in Myocyte Maintains Muscle Regeneration in Duchenne Muscular Dystrophy
Source: J Cachexia Sarcopenia Muscle. 2025 Apr 21;16(2):e13807. doi: 10.1002/jcsm.13807 (PMC12011492; doi:10.1002/jcsm.13807)

**Oestrogen receptor alpha in myocyte maintains muscle regeneration in Duchenne muscular dystrophy**

Xiaofei Huang^1,6^, Sijia Li^1,6^, Huna Wang^1^, Lei Zhao^2^, Xihua Li^2^, Shusheng Fan^1^, Wanting Hu^1^, Haowei Tong^1^, Guangyao Guo^1^, Dengqiu Xu^3^, Luyong Zhang^1,4^, Zhenzhou Jiang^1,5,7*^, Qinwei Yu^1*^

^1^New Drug Screening Center, Jiangsu Center for Pharmacodynamics Research and Evaluation, China Pharmaceutical University, Nanjing, China

^2^Department of Neurology, Children's Hospital of Fudan University, Shanghai, China

^3^Department of Hepatobiliary Surgery, Innovative Institute of Tumor Immunity and Medicine (ITIM), Anhui Province Key Laboratory of Tumor Immune Microenvironment and Immunotherapy, The First Affiliated Hospital of Anhui Medical University, Hefei, China.

^4^Center for Drug Research and Development, Guangdong Pharmaceutical University, Guangzhou, China

^5^Key Laboratory of Drug Quality Control and Pharmacovigilance, Ministry of Education, China Pharmaceutical University, Nanjing, China

^6^These authors contributed equally.

*Correspondence: No. 24, Tongjia Lane, Gulou District, Nanjing 210009, China.

Email: beaglejiang@cpu.edu.cn (Zhenzhou Jiang), yuqinwei7213@cpu.edu.cn (Qinwei Yu)

**Supplement western blotting**


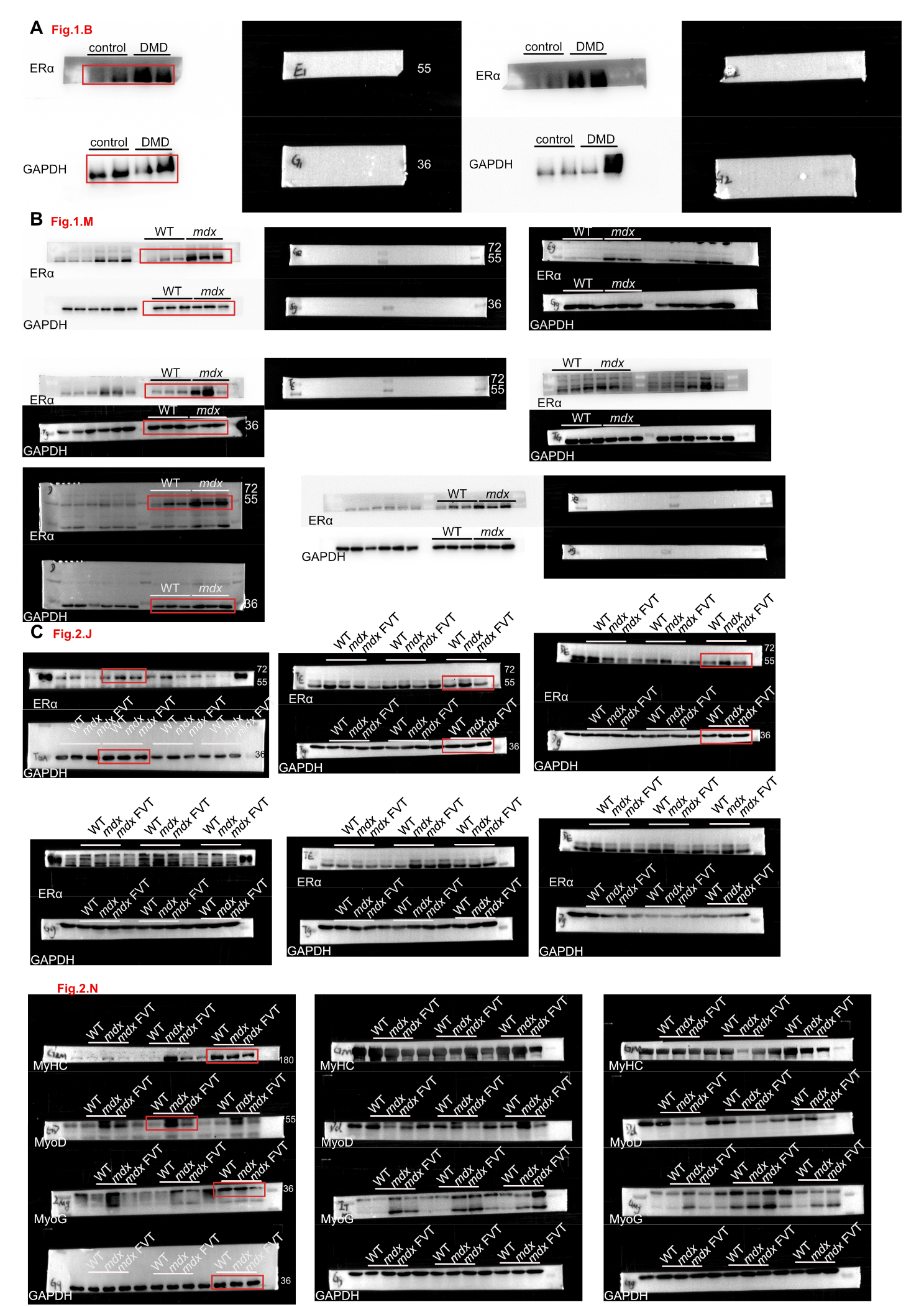

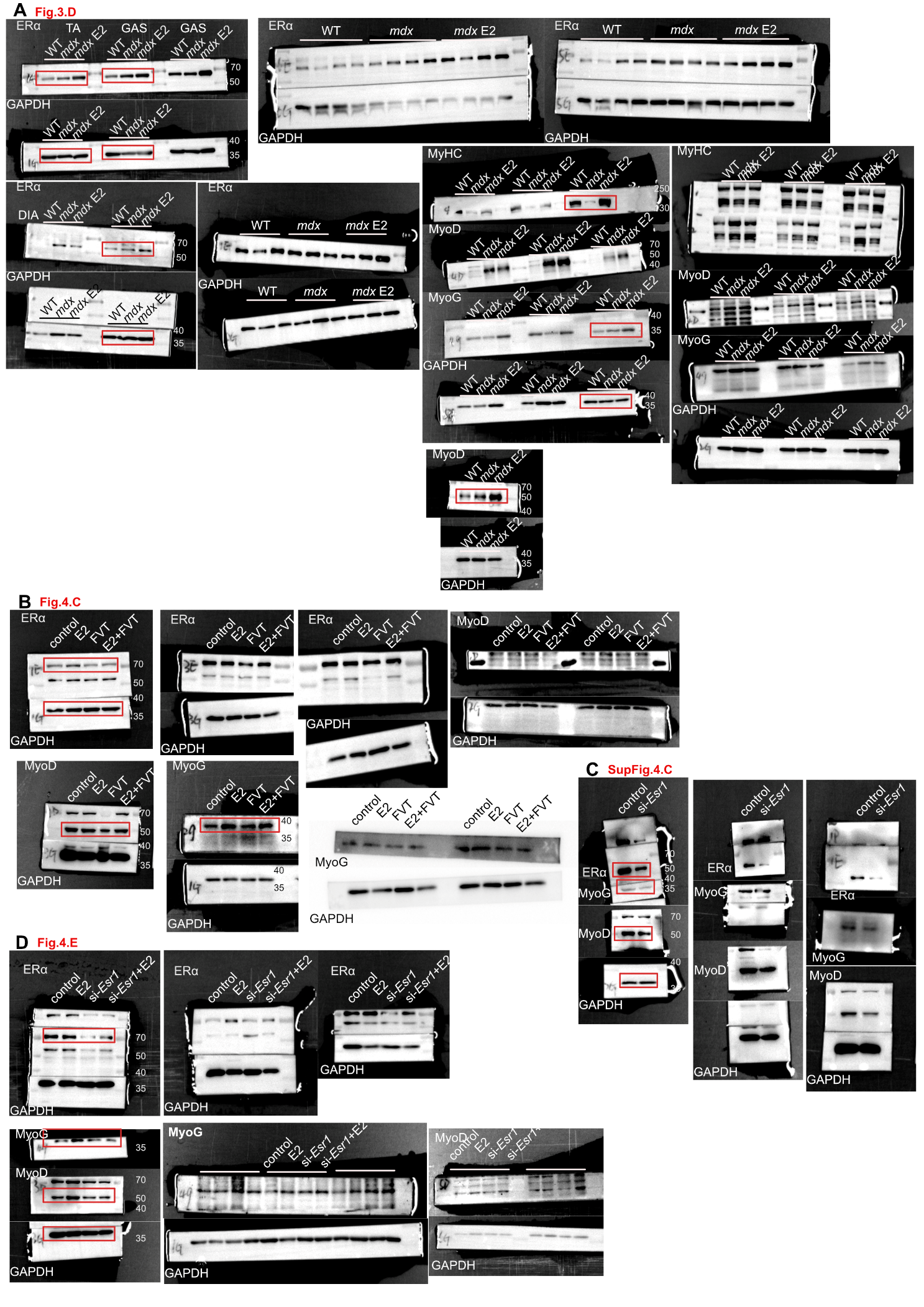

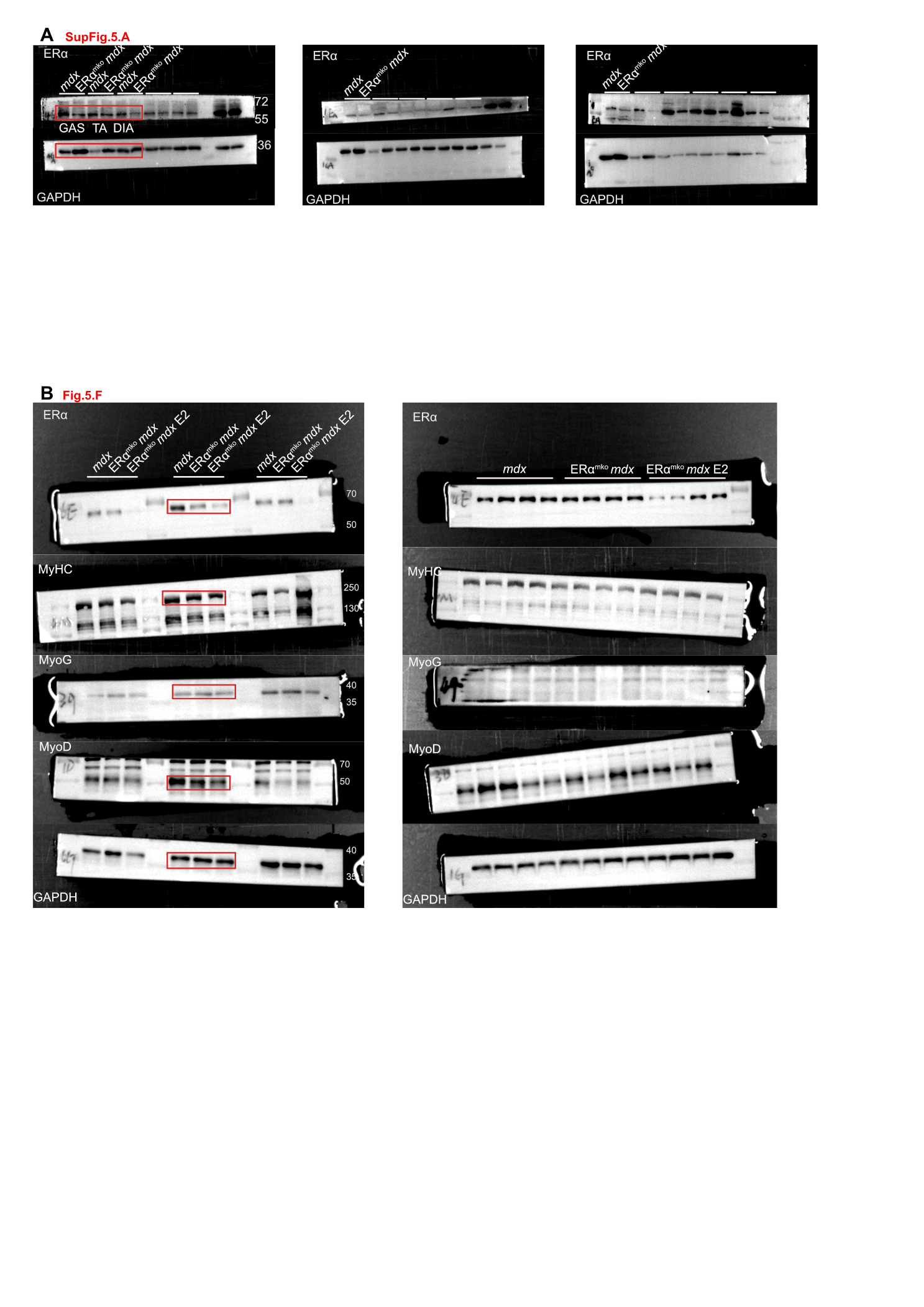


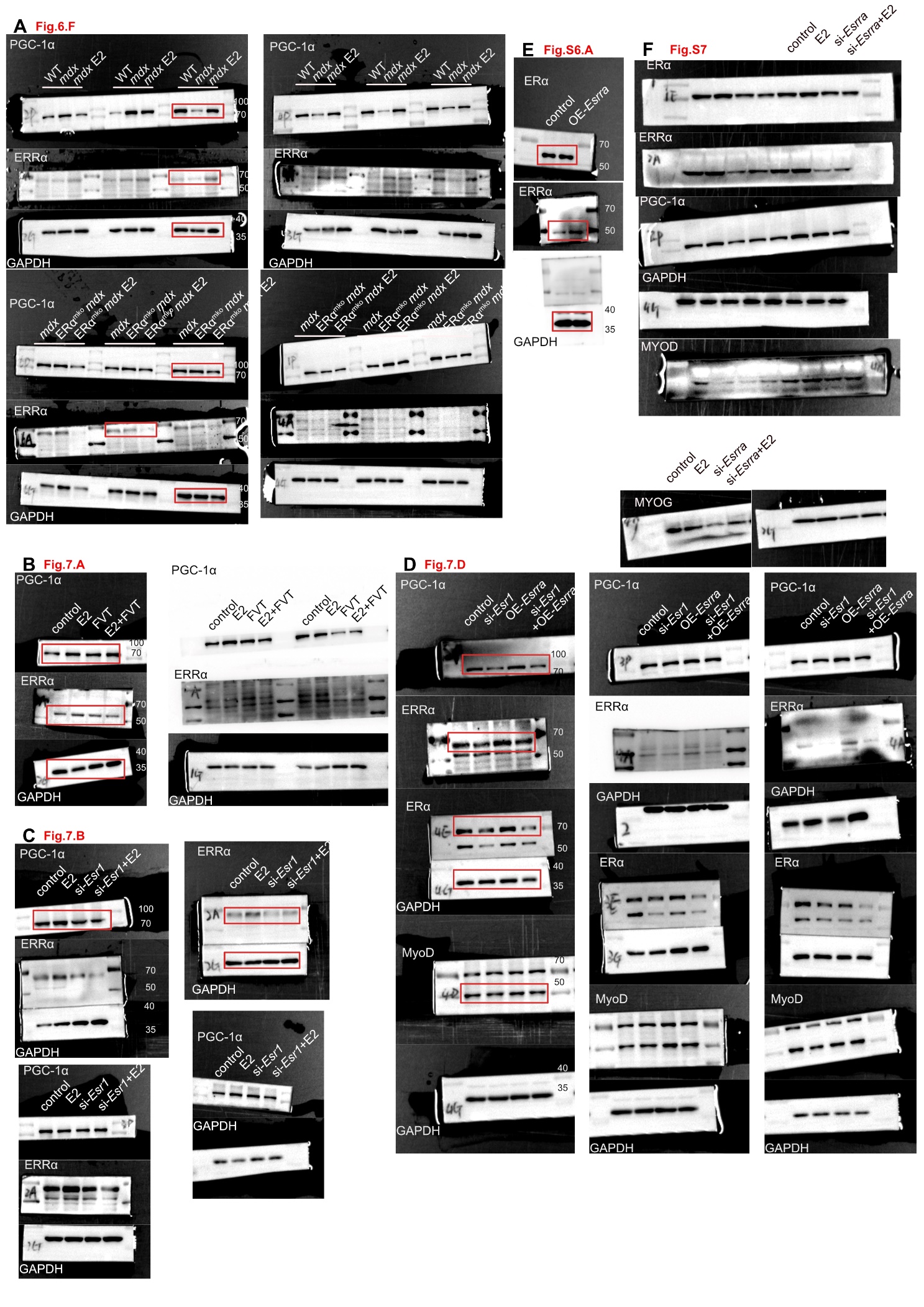

Supplement: Supplementary file 2 — Data S1 Supporting Information. [file JCSM-16-e13807-s001.docx]
